# Motion direction biases around the clock: Learned and in-built direction priors pull perception and pursuit apart

**DOI:** 10.1167/jov.26.3.11

**Published:** 2026-03-23

**Authors:** Liubov Ardasheva, Anna Montagnini

**Affiliations:** 1 Institut de Neurosciences de la Timone, CNRS and Aix-Marseille Université, Marseille, France

**Keywords:** tracking eye movements, motion perception, bayesian inference, naturalistic biases, perception and eye movements relation

## Abstract

Visual motion perception and pursuit eye movements rely on integrating uncertain sensory input with prior knowledge. Previous work has extensively investigated motion perception biases from experience-related or innate priors. In parallel, since Eileen Kowler's pioneering work, anticipatory smooth eye movements have been studied as an indicator of cognitive expectations. However, whether perception and eye movements rely on the same priors and computational operations (e.g., Bayesian reliability-based integration) remains only partly understood. Additionally, the role of natural directional biases in two-dimensional space (e.g., cardinal preferences) and their interaction with immediate motion expectations have not been explored. To address these questions, we measured smooth pursuit and direction estimations in human volunteers tracking random dot kinematograms with a proportion of coherent dots (5%, 15%, or 40%) moving in one of 16 directions between −180° and +180° across three sessions: one with uniformly distributed directions and two including a specific directional bias. Under high uncertainty, inaccurate direction estimations systematically avoided the most frequent direction in biased sessions, contrary to the Bayesian attraction-to-prior predictions, and generally favored cardinal directions. In contrast, eye movements agreed with the attraction-to-prior effect: Anticipatory pursuit roughly aligned with expected directions, and early pursuit acceleration was enhanced when stimulus direction matched expectation. These findings highlight a dissociation between perception and pursuit in directional biases induced across time scales in two-dimensional space. This suggests that the two systems either rely on partly different internal models or use shared priors differently, pointing to a layered, task-dependent organization of motion inference in the brain.

## Introduction

In everyday life, we rely on our vision to navigate, act, and predict future events, and often we have to do all this quickly and with uncertain and partly ambiguous information. This is especially true when processing visual motion and interacting with dynamic environments. In order to catch a ball, cross a busy highway, or just walk through a crowd without bumping into anyone, we have to rapidly interpret multiple and partly occluded complex sensory cues, to attribute them to the same or different objects in the visual scene, and eventually to form robust estimates about what is happening around us and act accordingly.

What guides our interpretations and actions when the visual dynamic scene is uncertain? An insightful way to investigate this is through visual motion estimation and eye tracking tasks. Those tasks may require discriminating (Dakin, Apthorp, & Alais, 2010; Mukherjee, Battifarano, Simoncini, & Osborne, 2015) or, more seldom, estimating (e.g., Chalk, Seitz, & Seriès, 2010) the direction (and/or speed) of a moving stimulus—forcing our perception to operate under uncertainty and commit to a decision. Alternatively (and only rarely in the same studies, unfortunately), researchers have analyzed different phases of voluntary tracking eye movements (saccades and smooth pursuit) (e.g., Goettker & Gegenfurtner, 2021; Orban De Xivry & Lefèvre, 2007). In both cases, previous findings have demonstrated and attempted to model the fact that perception and movements (including eye movements) are shaped not only by immediate sensory evidence but also by prior knowledge, learned expectations, and inherent biases rooted in both environmental regularities and neural architecture (e.g., to cite only a few, Harrison, Bays, & Rideaux, 2023; Kim, Park, & Lee, 2019; Körding & Wolpert, 2004; Kowler, 2011; Kowler, Rubinstein, Santos, & Wang, 2019; Krauzlis & Adler, 2001; Stocker & Simoncelli, 2006). This ability to integrate current input with past experience and longer-lasting beliefs is fundamental to how we perceive, decide, and act.

Despite a vast literature addressing these questions at multiple levels, several core issues remain only partially resolved. For example, how are priors and immediate sensory information integrated over time to control movements and perception? And across space, or along different motion directions? How do different priors, such as those shaped by experiences on different time scales, interact? And do perception and action rely on the same mechanisms for integrating information, or are they at least partially dissociated?

Answering these questions can shed light on broader mechanisms of inference and learning. By understanding how motion perception and eye movements operate under uncertainty, we gain insight into how the brain integrates sensory signals with memory and expectation to guide behavior. Eileen Kowler has been a pioneer in this approach, and her ingenious way to design simple and thoughtful experiments to directly address big questions has paved the way for flourishing research.

A relevant theoretical framework addressing the question of information integration in a principled way is the Bayesian approach to perceptual and sensorimotor decision making. According to this probabilistic model, the brain combines noisy sensory evidence (likelihood) with expectations derived from past experience or in-built beliefs (priors) to form a posterior estimate that informs both perceptual judgments and motor behavior (e.g., Körding & Wolpert, 2004; Mao & Stocker, 2024). Numerous studies support this view, showing, for example, that smooth pursuit eye movements can be biased in the direction of a learned prior (attraction-to-prior), especially when the current input is weak (Darlington, Beck, & Lisberger, 2018; Deravet, Blohm, De Xivry, & Lefèvre, 2018; Kowler et al., 2019; Rubinstein, Singh, & Kowler, 2024). For example, Darlington et al. (2018) (see also Yang, Lee, & Lisberger, 2012) demonstrated that a monkey's early visually guided smooth pursuit is influenced by prior stimulus-speed history, with the strongest effects emerging when sensory signals are degraded (e.g., low luminance contrast), consistent with reliability-weighted integration of prior and likelihood information. Similarly, Deravet et al. (2018) found that human pursuit eye velocity reflects a weighted combination of current sensory input and short-term memory of previous motion speed, and that this integration depends on the relative reliability of the two sources. Previous studies have proposed that pursuit behavior during the transient blanking of a visual moving target can be simulated by a dynamic probabilistic inference model (Bogadhi, Montagnini, Mamassian, Perrinet, & Masson, 2011; Bogadhi, Montagnini, & Masson, 2013; Orban De Xivry, Coppe, Blohm, & Lefèvre, 2013). In addition, the seminal work of Kowler on anticipatory smooth pursuit (Kowler, Martins, & Pavel, 1984; Kowler & Steinman, 1979a; Kowler & Steinman, 1979b) demonstrated that anticipatory pursuit is influenced by cognitive factors such as expectations and predictive knowledge, seeding a whole line of research that further characterized the sensitivity of this behavioral measure to environmental regularities and expectations about motion direction and speed (Badler & Heinen, 2006; Boman & Hotson, 1988; Carneiro Morita, Souto, Masson, & Montagnini, 2025; Damasse, Perrinet, Madelain, & Montagnini, 2018; De Hemptinne, Lefèvre, & Missal, 2006; De Hemptinne, Lèfevre, & Missal, 2008; Miyamoto, Hirata, Katoh, Miura, & Ono, 2021; Pasturel, Montagnini, & Perrinet, 2020; Santos & Kowler, 2017; Wu, Rothwell, Spering, & Montagnini, 2021). As nicely reviewed by Kowler et al. (2019) (for reviews, see also Barnes, 2008; Fukushima, Fukushima, Warabi, & Barnes, 2013), these studies converge toward the view that anticipatory smooth pursuit reflects the influence of internal dynamic priors for visual motion properties. Altogether, these studies provide compelling evidence that the oculomotor system, like perceptual systems, incorporates prior knowledge, whether based on short- or longer-term information, and does it in a Bayesian manner to optimize behavior under uncertainty (Knill & Pouget, 2004; Körding & Wolpert, 2004; Montagnini, Mamassian, Perrinet, Castet, & Masson, 2007; Pouget, Beck, Ma, & Latham, 2013).

Interestingly, a separate line of research has recently challenged the idea that motion perception always integrates priors in an *attraction-to-prior*, Bayesian fashion. In a previous study of our group, observers exhibited systematic repulsion *away* from the expected direction in a two-direction random dot kinematogram (RDK) motion task (Wu et al., 2021). This finding was even more surprising considering that, in the same study, robust anticipatory eye movements *toward* the expected direction have been reported. However, repulsion effects have been observed in many previous studies. In motion perception tasks, perceptual reports were biased slightly away from a reference direction (Rauber & Treue, 1998); in tasks involving motion judgments for many different directions, errors in favor of the diametrically opposite direction were more frequent than others when the stimulus was poorly visible (Bae & Luck 2022; Chetverikov & Jehee, 2023). These responses could not be attributed only to memory lapses or random guesses but they reflected systematic and coherence-dependent misalignment in perception (Jazayeri & Movshon, 2007; Ma & Jazayeri, 2014; Stocker & Simoncelli, 2006). In addition, the repulsion could not be simply explained by low-level sensory adaptation or as a direct consequence of eye movements (Wu et al., 2021; but see Miyamoto, Numasawa, & Ono, 2022).

In parallel, a large body of literature has documented both attractive and repulsive perceptual biases at shorter time scales, typically described as serial dependence. In these studies, the percept on a given trial is systematically pulled toward (attraction) or pushed away from (repulsion) recently seen stimuli (Bosch, Fritsche, Ehinger, & de Lange, 2020; Fischer & Whitney, 2014; Moon & Kwon, 2022). These effects have been observed across a variety of perceptual features, including orientation (Fischer & Whitney, 2014; Fritsche, Mostert, & De Lange, 2017), numerosity (Anobile, Cicchini, & Burr, 2014; Cicchini, Mikellidou, & Burr, 2017), facial identity (Liberman, Fischer, & Whitney, 2014), and motion direction (Kwon & Knill, 2013). The direction and magnitude of the bias depend on task demands, uncertainty, stimulus similarity, attention, and temporal context. For example, Fritsche et al. (2017) showed that repulsion is more likely under high sensory noise or when memory load is emphasized, whereas attraction dominates under conditions of low uncertainty and continuous viewing. Zhang and Alais (2020) further demonstrated that both attraction and repulsion can co-exist, driven respectively by previous perceptual choices and motor responses (see also Moon & Kwon, 2022).

This growing body of work has established that the integration of prior information by the brain is dynamic and context dependent, often shaped by the time scale, task demands, and computational level of processing involved. However, much of this literature focuses on immediate past trials and local stimulus statistics. In contrast, our interest lies in understanding how longer-lasting environmental and experience-related priors influence perception and action and whether attraction and repulsion can result from the same global structure and how they are differentiated across behavioral systems. For general reviews on examples of dissociations between the perceptual and oculomotor accounts of visual motion processing, see Spering and Montagnini (2011) and two articles by Spering and Carrasco (Carrasco & Spering, 2024; Spering & Carrasco, 2015).

To explore this, we turn to smooth pursuit eye movements as a readout of prior (i.e., during the anticipatory phase) and posterior (during the visually guided phase) estimate of visual motion. Unlike perceptual reports, which require explicit decisions, pursuit provides a continuous, low-level measure of motion processing that unfolds in time and is sensitive to both prior expectations and sensory uncertainty. By examining how pursuit adapts to directional statistics and how that adaptation compares to perceptual responses we aimed at disentangling how different systems access, represent, and integrate prior knowledge.

This study builds directly on two previous works, one by our own group (Wu et al., 2021), and the other by Rubinstein et al. (2024). In both of these studies, participants observed RDKs with variable sensory reliability, and the frequency of occurrence of visual motion directions was manipulated across the experimental sessions. The effects of such manipulation were analyzed on both pursuit and perception (Wu et al., 2021) or on different phases of pursuit (Rubinstein et al., 2024).

Wu et al. (2021) presented observers with RDK motion stimuli with global motion direction drawn repeatedly from a biased distribution (right/left binomial distribution parameterized by the probability *p* of rightward motion occurrence) and found that anticipatory eye movements were increasingly enhanced, with *p*, to the most frequent direction. Surprisingly, for low-coherence RDK stimuli, perceptual reports were instead relatively repelled away from the expected direction, suggesting that pursuit and perception may rely on distinct prior representations or differ in how they integrate the prior with uncertain sensory evidence. This study offered a compelling dissociation: Anticipatory eye movements reflected attraction to the prior, whereas perception showed repulsion during the same task. However, the generalizability of the findings of Wu and collaborators is limited by the fact that we only tested unidimensional (horizontal) motion. In the present study, we decided to investigate the effect of a directional bias across the entire plane (or, in other terms, across 360°).

Rubinstein et al. (2024) did not analyze perceptual motion estimation, but they demonstrated that not only does anticipatory pursuit adjust to the mean direction of past motion but it also depends on its variability. Their work builds on decades of evidence, from Kowler's group and others, showing that anticipatory pursuit is highly sensitive to statistical regularities in motion and reflects an internal model of environmental structure (Damasse et al., 2018; Kowler, 1989; Kowler & Steinman, 1979b; Pasturel et al., 2020; Santos & Kowler, 2017). In their study, Rubinstein et al. (2024) also reported that early visually guided pursuit responses are initially aligned with the prior, but, as time unfolds, they become increasingly dominated by the sensory evidence, thus suggesting a transition from prior-driven to sensory-driven guidance. Note, however, that the predictions of a Bayesian integration model were only partially validated by their results. Rubinstein et al. (2024) also extended the analysis of pursuit to non-horizontal axes, showing that both anticipatory and visually guided eye movements adapt flexibly to directional priors presented along different trajectories. Their design was intended to isolate the effects of the prior and sensory likelihood from naturalistic biases, which allowed them to demonstrate clear anticipatory adaptation. Yet, because the stimulus set did not cover all-around-the-clock motion conditions (i.e., in a given trial the possible directions were limited to an angular cone around the mean direction), the study did not address the full range of possible adaptations, particularly how induced priors might interact with pre-existing environmental or naturalistic biases, such as the well-documented preference for cardinal motion directions (Andrews & Schluppeck, 2000; Ezzo, Winawer, Carrasco, & Rokers, 2023; Girshick, Landy, & Simoncelli, 2011).

The time-course analysis of Rubinstein and collaborators helps define a three-stage framework for smooth pursuit analysis: anticipatory pursuit (before and around stimulus onset); early, open-loop visually guided pursuit (100–200 ms after stimulus motion onset); and closed-loop steady-state pursuit (beyond ∼300 ms). During anticipation, eye movements are supposed to be affected exclusively by non-visual (prior-related) information. During the early open-loop stage, eye velocity is influenced by visual input, but the system does not yet incorporate feedback about movement of the eye, and the influence of priors is presumably still important. This contrasts with the closed-loop stage that follows, where pursuit is continuously adjusted, based on both retinal slip and efferent signals about eye motion, and the longer-term prior information becomes less relevant (Bogadhi et al., 2013). This distinction is critical, as Bayesian integration of prior and likelihood may be expressed differently across these stages, as shown by Rubinstein et al. (2024).

To investigate how long-term motion direction priors shape both perception and action, we designed a task that jointly measured smooth pursuit and perceptual direction estimation under varying probabilistic conditions of visual motion direction occurrence and varying sensory uncertainty. Specifically, across three experimental sessions, participants were exposed to RDK motion directions drawn from a uniform angular distribution (Session 1, unbiased) or distributions biased toward a cardinal direction (Session 2, 0°, horizontal rightward biased) or an oblique direction (Session 3, −135°, up-left biased). Motion coherence was manipulated randomly, across trials, to vary sensory uncertainty, and participants were instructed both to visually track the stimulus and to report its perceived direction by drawing it with a mouse. Eye movements were recorded throughout.

The choice of frequent motion directions in the biased sessions was motivated as follows. The 0° (rightward) direction served as a benchmark for testing responses to motion along a naturally dominant, environmentally frequent axis (and one already tested in Wu et al., 2021), whereas the −135° (up-left) direction allowed us to probe whether any possible effect of prior attraction, repulsion, and Bayesian updating generalizes to less common, oblique trajectories. A secondary aim of our study was also to test whether natural biases favoring cardinal directions (Ezzo et al., 2023) modulate the strength or direction of behavioral responses in the unbiased experimental context and how they interact with imposed directional biases in the biased sessions. In summary, we replicated the horizontal motion paradigm used by Wu et al. (2021) while extending its scope in line with the multidirectional framework of Rubinstein et al. (2024) and explicitly testing how environmental priors interact with experimentally induced ones to influence motion perception and smooth tracking eye movements.

Our findings reveal common features but also a dissociation between direction estimations of noisy stimuli and anticipatory eye movements in how they reflect prior knowledge. First, in the unbiased Session 1, where motion directions were sampled uniformly, we observed non-uniform distributions of responses in both behavioral measures, with strong biases favoring cardinal directions in perceptual responses under high uncertainty and less so for eye movements. In biased sessions, perceptual estimates in low-coherence trials showed an overall *avoidance* of the most frequently presented direction, indicating a bias away from the prior, however not exactly a repulsion toward the opposite direction. Overall, perceptual choices remained dominated by cardinal biases throughout, although in interaction with the experience-driven modulations, suggesting an important role of long-standing (in-built) internal priors for motion direction estimate.

In contrast, across sessions, anticipatory pursuit aligned to biased direction distributions (yet this effect was significant only in rightward Session 2, where motion was biased toward a cardinal direction). Interestingly, only early open-loop accelerating pursuit showed consistent and robust effects of the experimental direction prior, in line with Bayesian integration of prior and likelihood, whereas steady-state pursuit showed no reliable modulation by session, but only by stimulus coherence.

Taken together, these results underscore the layered and complex nature of predictive processing across perceptual and motor systems. They reveal that cardinal preferences are deeply embedded in both anticipatory and perceptual domains, that early acceleration reflects flexible Bayesian updating, and that distinct stages of behavior capture different signatures of prior and likelihood integration. These findings challenge the idea of a single, unified Bayesian decoder and instead support a modular, time scale- and task-sensitive architecture for integrating prior knowledge across systems.

## Methods

### Participants

A total of 21 healthy adults (12 females; mean age = 26 years, *SD* = 3.95 years) with normal or corrected-to-normal vision participated in the study. All participants provided informed consent in accordance with the tenets of the Declaration of Helsinki, and the study was approved by the national ethics committee (CPP VisAct 2022-A01584-39). Each participant received a payment of 30 euros in total, for three sessions, in gift vouchers.

### Apparatus

The experiment was conducted in a room with low illumination, with participants seated at a distance of 57 cm from a 24-inch LCD monitor (Display++; Cambridge Research Systems, Rochester, UK; 1920 × 1080-pixel resolution and 85-Hz refresh rate). The stimulus presentation and data collection were controlled using MATLAB 2018 (MathWorks, Natick, MA) with the Psychophysics Toolbox extensions (Brainard, 1997; Pelli, 1997). Eye movements were recorded using an EyeLink 1000 tower-mounted eye-tracker (SR Research, Ottawa, ON, Canada), with a sampling rate of 500 Hz.

### Stimuli

The stimulus was a RDK consisting of approximately 470 white dots (density, 1.5 dots/deg^2^; luminance, 98 cd/m^2^) uniformly distributed within a circular aperture of 10° radius in the center of the screen on a gray background (luminance, 22 cd/m^2^). Each dot, with a diameter of 0.14°, moved at a constant speed of 10°/s. Dots were classified as either signal or noise, with labels updated and randomly reassigned every four frames (47 ms). Signal dots always moved in one of the 16 global motion directions (RDKdir, randomized across trials), whereas noise dots moved in random directions unrelated to the global direction and had unlimited lifetimes. In compliance with label reshuffling, a new random direction was assigned to all noise dots every four frames. When a dot moved outside the aperture, it re-entered from the opposite side. The coherence levels, which determined the proportion of signal dots moving coherently in the global motion direction (while noise dots moved randomly), were set at 5%, 15%, and 40%. The global motion of the stimulus was in one of 16 possible motion directions, separated by 22.5°, covering the entire 360° space and including the cardinal and main diagonal directions. The stimulus duration was 1000 ms in each trial. The participant's head was stabilized using a chin-and-forehead rest.

The particular stimulus design (RDK with signal- and noise-dots populations) was a replica of the one implemented in a previous work of our group (Wu et al., 2021). It is important to notice that this kind of stimuli can induce a sensation of *motion transparency* (see Schütz, Braun, Movshon, & Gegenfurtner, 2010), with a clear segregation between a coherently moving surface and a random-motion surface. Motion transparency could also facilitate a surface-based attentional selection of the coherent signal dots and enhance eye-tracking accuracy (Schütz et al., 2010). Note that, although motion transparency can in principle influence both direction estimation and oculomotor tracking, this possibility does not invalidate the present study, based on the comparison of perception and eye movements across experimental conditions using the same RDK stimulus.

### Procedure

Participants completed three separate sessions on three different days, each defined by a specific probability distribution of RDK motion directions. The order of the three sessions was randomized across participants. In unbiased Session 1 (480 trials), the motion direction of signal dots was uniformly distributed, with equal probability across 16 possible directions spaced at 22.5° intervals around the aperture (0°, 22.5°, 45°, …). Sessions 2 and 3 consisted of 474 trials each and were structured to introduce directional biases. Within each session, the bias was the same for each coherence level. For all coherence levels, most trials featured motion toward a single overrepresented (“frequent”) direction: 0° (rightward) in Session 2 and −135° (up-left) in Session 3. Frequent-direction trials made up approximately 60% of the session, and the remaining ∼40% of trials were distributed evenly across all other directions. Session order was randomized across participants.

**Figure 1.**
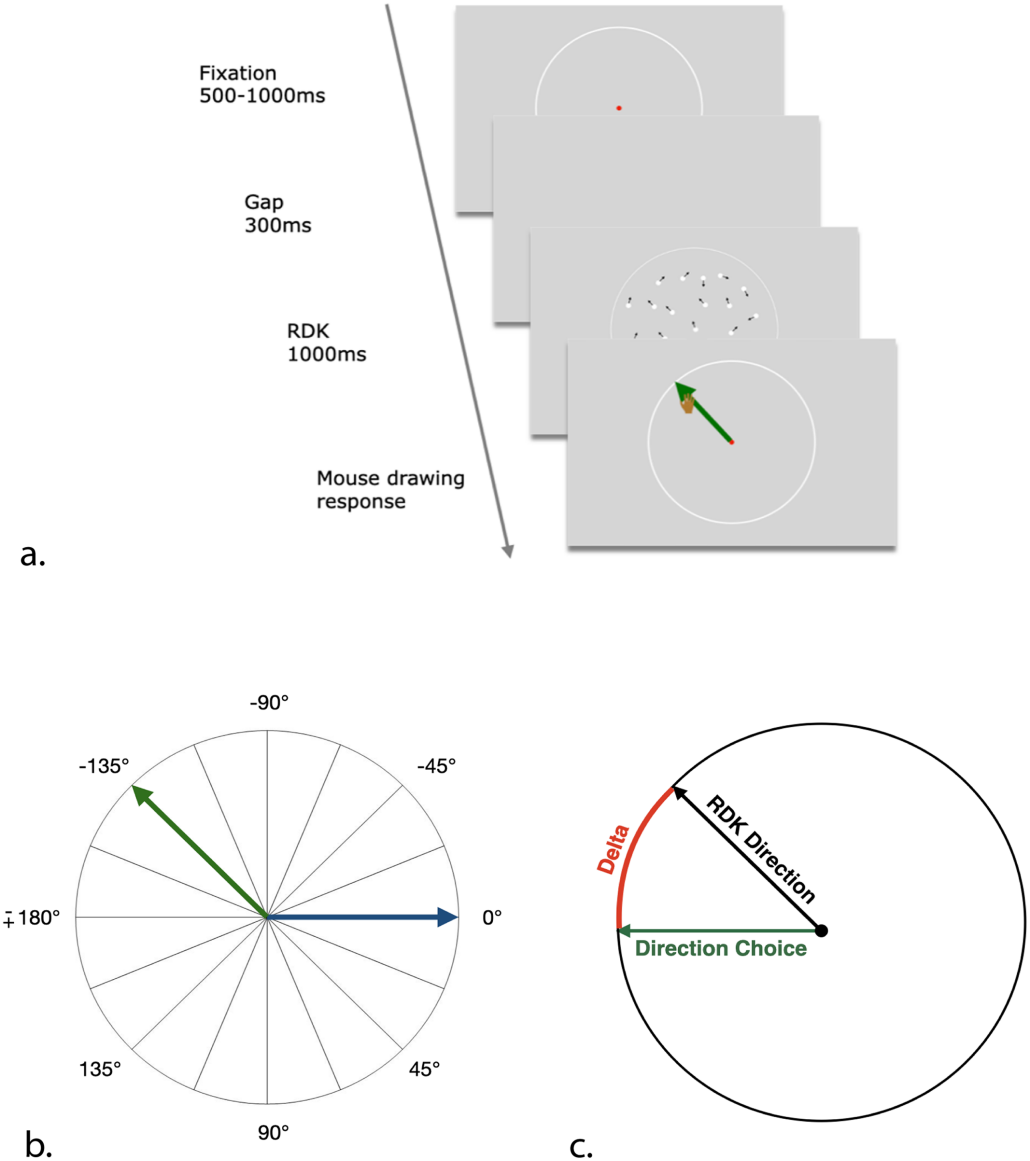
Experimental design, angular layout with session-specific motion biases, and response measurement. (**a**) Trial structure. Each trial began with central fixation (500–1000 ms), followed by a 300-ms gap and a 1000-ms presentation of a RDK moving coherently in one of 16 possible directions. Participants reported the perceived motion direction by freely drawing with the mouse, after which the computer displayed a straight vector based on their input, which could have been adjusted if needed. (**b**) Angle layout and directional priors across sessions. In all figures, 0° indicates rightward motion, positive values correspond to downward directions, and negative values indicate upward directions. In Session 1, directions were uniformly sampled (unbiased); in Session 2, frequent motion was rightward (0°, *blue*); and, in Session 3, frequent motion was up-left (−135°, *green*). (**c**) Angular error (*delta*) was computed as the signed difference between the actual RDK direction and the reported direction, shown as the shortest arc on the unit circle.

Each trial (see Figure 1a) began with the presentation of a central fixation dot for a randomly determined duration between 500 and 1000 ms. Participants were required to maintain their gaze within a predefined invisible spatial window (40 × 40 pixels) around the fixation point. Following the fixation period, a 300-ms gap was introduced, during which the screen remained blank. This gap, with fully predictable temporal duration, was specifically intended to facilitate anticipatory eye movements (e.g., Damasse et al., 2018).

Subsequently, the RDK stimulus was displayed for 1000 ms. Participants were instructed to observe the moving dots, tracking the perceived global motion with their gaze, and to estimate the direction of motion. After the stimulus presentation, participants responded by drawing the perceived motion direction using a computer mouse. The computer would then draw a straight line displaying the chosen angle direction. If necessary, they could adjust their drawn direction by double-clicking within the aperture. When they were satisfied with their response, participants confirmed their choice by pressing the space bar. The mouse movements were not recorded except for the final chosen point on the aperture.

### Eye-tracking and gaze monitoring

Eye movements were recorded continuously during each trial of the experiment. The EyeLink 1000+ tracked each participant's right-eye position to ensure that fixation was maintained during the initial fixation period and to record smooth pursuit movements during the RDK presentation. After the initial nine-point gaze calibration procedure, drift correction was performed every 50 trials (and a new calibration was run, if needed) to make sure that an accurate gaze calibration was maintained all throughout the experimental session.

## Measures for data analysis

### Behavioral measures

Two primary behavioral measures were calculated: direction choice and angular error (delta).

#### Direction choice

This measure captured the direction reported by the participant as their perceived the direction of motion. During each trial, participants responded by either clicking on the circular contour of the RDK or by drawing a line from the center outward using the mouse. Upon receiving the initial input, the system displayed a straight line from the center of the aperture to the indicated point, representing the participant's selected direction of perceived motion. Participants were then allowed to adjust this line if needed. When they were satisfied with their response, participants confirmed their choice by pressing the space bar. The final orientation of the line, whether initially drawn or corrected, was recorded as a single angular value in degrees. This value reflected the participant's direction estimate and served as the basis for computing accuracy.

#### Angular error

To quantify direction estimation accuracy, we calculated the error (delta) as the angular difference between the direction of RDK global motion and the participant's reported direction. To classify response accuracy, we applied a bin-based approach aligned with the structure of the stimulus space, which was discretized into 16 equally spaced motion direction bins (each 22.5° wide, centered at 0°, 22.5°, 45°, …). Each bin extended symmetrically ±11.25° around its center. A participant's response was considered correct if it fell within this ±11.25° range around the actual motion direction. For example, if the RDK direction was 0°, responses between −11.25° and +11.25° were labeled as correct. Responses falling outside this ±11.25° range were classified and are referred to throughout the text as incorrect trials.

This bin-based classification ensured consistency with the angular resolution of the task and allowed us to accommodate small fluctuations in participants’ responses due to motor imprecision or fine perceptual variability. By defining correctness as falling within the exact bounds of the stimulus bin, the scheme avoided over-penalizing slight deviations near bin boundaries while maintaining a principled threshold tied to the experimental design. Additionally, responses falling within ±11.25° of the direction opposite to the target (i.e., 180° offset from the actual motion direction) were labeled as *opposite* errors. All remaining responses were classified as *general* errors. The rationale for distinguishing between opposite errors and general ones was not solely descriptive. These categories were defined in accordance with previous literature to allow for hypothesis-driven analysis of systematic perceptual distortions, such as strong repulsion effects, in motion direction estimation (Wu et al., 2021).

Because all stimuli and responses in our experiment were angular (measured in degrees), we used circular statistics to compute angular differences and means and variability measures. This avoided misinterpretation of circular data—for example, correctly treating the difference between 350° and 10° as 20°, rather than 340°. All computations, including binning and averaging, were conducted within a circular framework.

### Eye-movement measures

To analyze smooth-pursuit eye movements, raw eye position data were low-pass filtered using a second-order Butterworth filter with a 30-Hz cutoff. The filtered position signals were numerically differentiated to obtain velocity estimates. To isolate smooth pursuit, saccadic eye movements were identified using a velocity threshold method (Bahill, Clark, & Stark, 1975; Larsson, Nyström, Andersson, & Stridh, 2015; Nyström & Holmqvist, 2010) and excluded by replacing saccade samples with Not a Number (NaN) values.

We extracted eye velocity metrics from three distinct temporal windows corresponding to different functional stages of the pursuit response: (1) anticipatory movements, (2) early acceleration phase, and (3) steady-state pursuit. Projected eye velocity was computed by projecting the two-dimensional eye velocity vector onto the direction of stimulus motion. Specifically, we calculated the dot product between the eye velocity vector (*V_x_*, *V_y_*) and the unit vector in the direction of stimulus motion (cosθ, sinθ), where θ is the motion direction in radians:

$$V_{proj}=V_{x}\cdot \;\cos\theta +V_{y}\cdot \sin\theta$$

Here, *V_x_* and *V_y_* are the horizontal and vertical eye velocities (in degrees per second), and θ is the direction of stimulus motion (in radians). The resulting *V_proj_* provides a signed scalar value representing how strongly the eye velocity aligns with the direction of RDK global motion.

#### Anticipatory phase (−50 ms to 100 ms around stimulus onset)

To investigate whether smooth pursuit eye movements adapted to repeated exposure to a specific motion direction, we first analyzed anticipatory pursuit, defined as the smooth eye movement response occurring before and shortly after the onset of the moving stimulus, before the typical visuomotor latency of about 100 to 120 ms (Carl & Gellman, 1987). This analysis was motivated by previous findings (Damasse et al., 2018; Kowler et al., 2019; Kowler & Steinman, 1979a; Kowler & Steinman, 1979b; Santos & Kowler, 2017), which demonstrated that anticipatory smooth eye movements often reflect learned motion expectations, in particular when stimulus direction (Damasse et al., 2018; Santos & Kowler, 2017) or speed (Carneiro Morita et al., 2025) is probabilistically biased. Under a Bayesian framework, the anticipatory phase is thought to reflect the influence of prior expectations in the absence of reliable sensory input (Rubinstein et al., 2024; Wu et al., 2021). In particular, if a specific motion direction is frequently presented, participants may begin to drift their gaze in that direction before the stimulus appears, based on their internal prediction. Here, we assessed this prediction by comparing anticipatory behavior toward the expected direction in the biased sessions (respectively 0° in rightward-biased Session 2 and −135° in up-left–biased Session 3) with the same behavior as measured in the baseline unbiased Session 1.

To quantify anticipatory velocity, we computed the mean horizontal and vertical components of the right-eye velocity in the anticipatory window. These components were used to calculate the anticipation amplitude and the anticipation angle (movement direction). Trials with extreme anticipatory velocity were excluded to remove residual saccades and noise. Specifically, we excluded trials with anticipation amplitudes above 2.5°/s (likely to include unmarked saccades) or below 0.25°/s (indicative of noise or unstable fixation). This amplitude-based criterion excluded fewer than 1% of trials (0.89%) across participants and conditions, preserving the vast majority of the data for analysis. We averaged the individual eye velocity in the anticipatory window, separately for each session, and we then selected trials where the direction of anticipation was corresponding to the biased directions for comparison—for example, bins of ±11.25° for 0° (i.e., rightward motion in Sessions 1 and 2) and for −135° (up-left motion in Sessions 1 and 3). Comparisons were performed using paired *t*-tests, Wilcoxon signed-rank tests (after Shapiro–Wilk normality checks), and Bayesian *t*-tests (*BF*_10_) with effect sizes (Cohen's *d*) reported. This allowed us to test whether repeated exposure to a motion direction enhanced anticipatory pursuit in that specific direction, consistent with predictive adaptation.

In addition, we examined the distribution of anticipatory movement directions by computing the normalized frequency of anticipatory responses across 16 angular bins (each spanning 22.5°). For each trial, the direction of the anticipatory eye movement (i.e., the angle of the velocity vector averaged across the anticipatory window) was assigned to one of the 16 bins. We then calculated, per subject and session, the proportion of trials falling into each direction bin, yielding a normalized anticipation frequency histogram. This analysis provided a more detailed view of how predictive movements were distributed across possible directions and allowed for assessing whether anticipatory movements clustered around the over-represented stimulus direction.

#### Acceleration phase (100 to 200 ms post-stimulus)

To capture the early visually guided acceleration phase of pursuit, we analyzed the mean eye acceleration between 100 and 200 ms after stimulus onset. This interval corresponds to the transition period during which the oculomotor system begins to respond to the actual motion stimulus but may still be influenced by prior expectations and by the combination of these with uncertain sensory input. Previous studies have shown that early pursuit gain can be modulated by the reliability of sensory input and by the statistical consistency of prior motion in non-human primates (Darlington et al., 2018).

Initial pursuit acceleration was estimated as follows. For each trial, we computed the difference between the mean projected eye velocity in two small temporal windows, 100 to 120 ms and 200 to 220 ms after RDK motion onset. These 20-ms bins were chosen to provide more stable estimates of velocity while accounting for variability in pursuit onset timing across individuals. To express acceleration in degrees per square second (°/s^2^), the velocity difference was divided by 0.1 second. Acceleration values were then averaged per subject, session, and coherence level to assess how early visually guided pursuit adapted across experimental conditions.

#### Steady-state pursuit (400 to 600 ms)

Finally, we assessed steady-state pursuit, defined as the average eye velocity between 400 and 600 ms after stimulus onset. This time window was chosen to capture the phase of smooth pursuit where eye velocity has stabilized and the gaze is tracking the stimulus in a sustained, visually guided manner. While early pursuit responses can reflect a mixture of preparatory signals and the onset of visually guided tracking, later phases are more directly shaped by the visual input itself (Rubinstein et al., 2024). Focusing on this stable window allowed us to assess pursuit behavior in an epoch where the motion signal was presumably fully available and reliably guided eye movements (Kowler & McKee, 1987; Lisberger, 2010).

We also expected that the steady-state eye velocity would increase with RDK coherence and that its correlation with direction estimations would improve, as steady-state pursuit is often closely aligned with motion judgments under clear sensory conditions (Barnes, 2008; Mukherjee et al., 2015; Spering & Montagnini, 2011). As in other pursuit analyses, we focused on the mean eye velocity projected in the direction of stimulus motion to quantify how well the eyes tracked the target during this stable phase.

### Serial dependence analysis measures

To assess serial dependence in perceptual responses, we analyzed how the current perceptual response (e.g., the direction estimation in trial *n*) was influenced by the difference between motion direction of the previous stimulus (trial *n* − 1) and motion direction in the current trial (*n*). We measured the perceptual angular error (delta) on trial *n* as a function of the angular difference in stimulus direction (rdkDir) between consecutive trials, rdkDir(*n*) − rdkDir(*n* − 1). All direction values were expressed as angular variables (in degrees) and transformed from the (0°, 360°) range into (−180°, 180°) using a circular wrap. This ensured that values near the boundaries (e.g., −179° and 179°) were treated as close rather than 360° apart. This analysis was conducted on unbiased Session 1 only, as it was the only condition without a systematic directional bias of the stimulus motion. By isolating this session, we aimed at evaluating whether perceptual reports were influenced by immediate trial history alone independently from global biases.

To evaluate serial dependence, we binned the difference in motion direction between consecutive trials (rdkDir_diff) into 22.5° steps, reflecting the 16 discrete motion directions used in the experiment. For each subject, we computed the mean delta (signed angular error, range −180° to 180°) as a function of rdkDir_diff. Because delta is an angular variable that does not follow a normal distribution, we analyzed it using the Watson–Williams one-way circular analysis of variance (ANOVA), implemented in both Python (with pycircstat) and MATLAB (CircStat toolbox) (Berens, 2009; Berens & Sinz, 2017). Both approaches yielded consistent results.

To claim that the response on the current trial was influenced by the RDK motion direction of the previous trial, we should observe a systematic modulation of angular error (delta) as a function of the angular difference between consecutive trials rdkDir(*n*) − rdkDir(*n* − 1). Based on serial dependence literature (e.g., Fischer & Whitney, 2014; Fritsche et al., 2017), this effect typically appears as a sigma-shaped (*S*-shaped) curve centered around 0°. Specifically:

• Attraction bias is indicated when delta systematically shifts toward the previous direction for small differences, showing a gentle negative-slope *S*-curve crossing zero.
• Repulsive bias, on the other hand, produces a positive-slope *S*-shaped curve, where errors are systematically pushed away from the prior direction.

These patterns have been documented in both orientation perception tasks (e.g., lines and gratings) (Fischer & Whitney, 2014) and motion direction tasks (Fritsche et al., 2017).

In addition to analyzing estimation error, we tested whether eye movement anticipation was influenced by recent motion history by analyzing the anticipation angle on a given trial as a function of the global motion direction (rdkDir) on the previous trial. Again, this analysis was performed only for unbiased Session 1. To assess this relationship at the group level, we fit a linear mixed-effects model predicting anticipation angle from the motion direction of the previous trial, with a random intercept for each subject. A systematic influence would be reflected as a linear relationship between the two variables.

## Results

### Behavioral results

Participants’ direction estimation performance varied as a function of RDK coherence and session-specific motion bias. Accuracy rates increased with coherence: In the unbiased Session 1, the proportion of correct responses rose from 17.7% at 5% coherence to 53.3% at 15% coherence and 79.4% at 40% coherence. In the rightward-biased Session 2, performance was slightly better, reaching 21.8%, 60.6%, and 84.0%, respectively. In contrast, the up-left–biased Session 3 showed the lowest performance at lower coherence levels (11.6% and 44.3%) but nearly matched the others at high coherence (77.5%).

These differences were confirmed by a generalized linear mixed-effects model (GLMM) predicting response correctness from coherence level and sessions (with random intercepts for participants). The model revealed significant effects of coherence (β = 7.72, *p* < 0.001) and session: Compared with the unbiased Session 1, performance was significantly higher in the rightward-biased Session 2 (β = 0.21, *p* < 0.001) and lower in the up-left–biased Session 3 (β = −0.45, *p* < 0.001). A significant interaction between coherence and Session 3 (β = 0.75, *p* = 0.002) indicated that the coherence-dependent improvement in accuracy was especially steep in that session.

### Direction estimation performance across different stimulus directions

**Figure 2.**
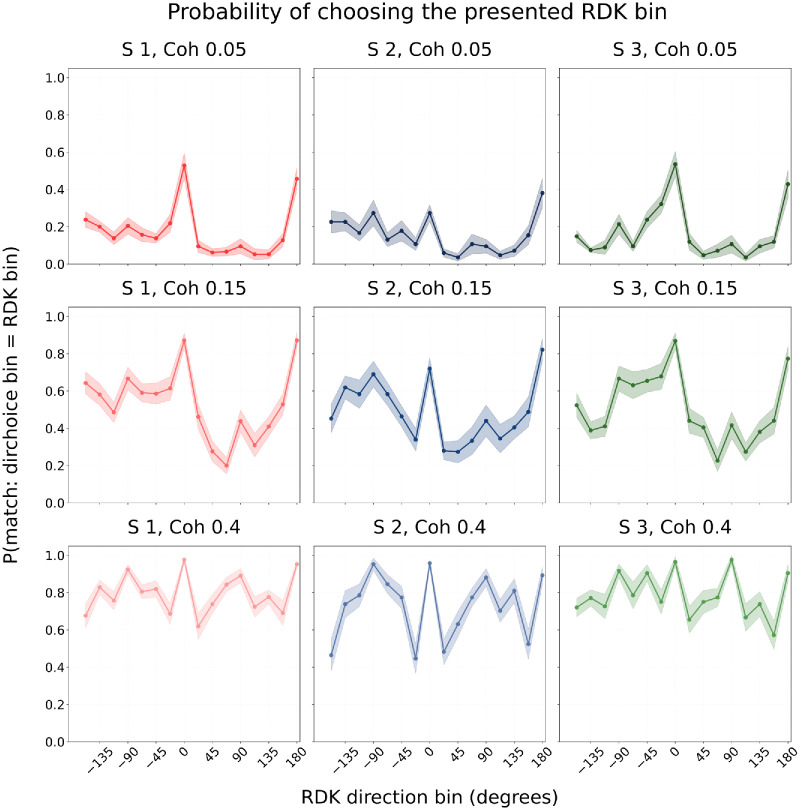
Probability of correct direction estimation across RDK direction bins. Mean probability across participants (±*SEM*) of reporting the true RDK direction (±11.25°) as a function of RDK direction, plotted separately for each coherence level (rows) in unbiased Session 1 (in *red*), rightward-biased Session 2 (in *blue*), and up-left–biased Session 3 (in *green*).

To characterize how direction estimation accuracy varied across motion directions, we examined the probability of selecting the correct RDK bin as a function of stimulus direction (Figure 2). In the unbiased Session 1, responses were not uniformly distributed, as participants were more accurate for cardinal directions than for any other directions. At intermediate coherence (15%), performance additionally appeared higher for directions in the upper hemifield, although this trend was less consistent and would require further targeted experiments to confirm.

In the rightward-biased Session 2, accuracy also varied strongly across directions. As expected, performance was relatively higher near the dominant direction (0°), but the overall cardinal advantage remained visible across coherences. In the up-left–biased Session 3, responses were again most accurate for cardinal directions; yet, at low and intermediate coherence levels participants showed particularly weak performance in the lower hemifield. At coherence 15%, responses in the upper hemifield appeared comparatively more accurate, echoing the descriptive trend seen in unbiased Session 1.

Because our experiment was not designed to specifically test hemifield asymmetries, we interpret these findings cautiously. Here, they serve to illustrate that response accuracy was systematically modulated by both stimulus coherence and direction, with robust advantages for cardinal directions and suggestive, but as yet inconclusive, evidence of an upper-hemifield benefit at intermediate coherence. Although these analyses reveal clear direction- and coherence-dependent patterns in response accuracy, they do not capture the nature of participants’ errors. To address this, we next turned to angular error distributions, which show how perceived motion directions deviated from the true stimulus.

#### Directional error distributions

**Figure 3.**
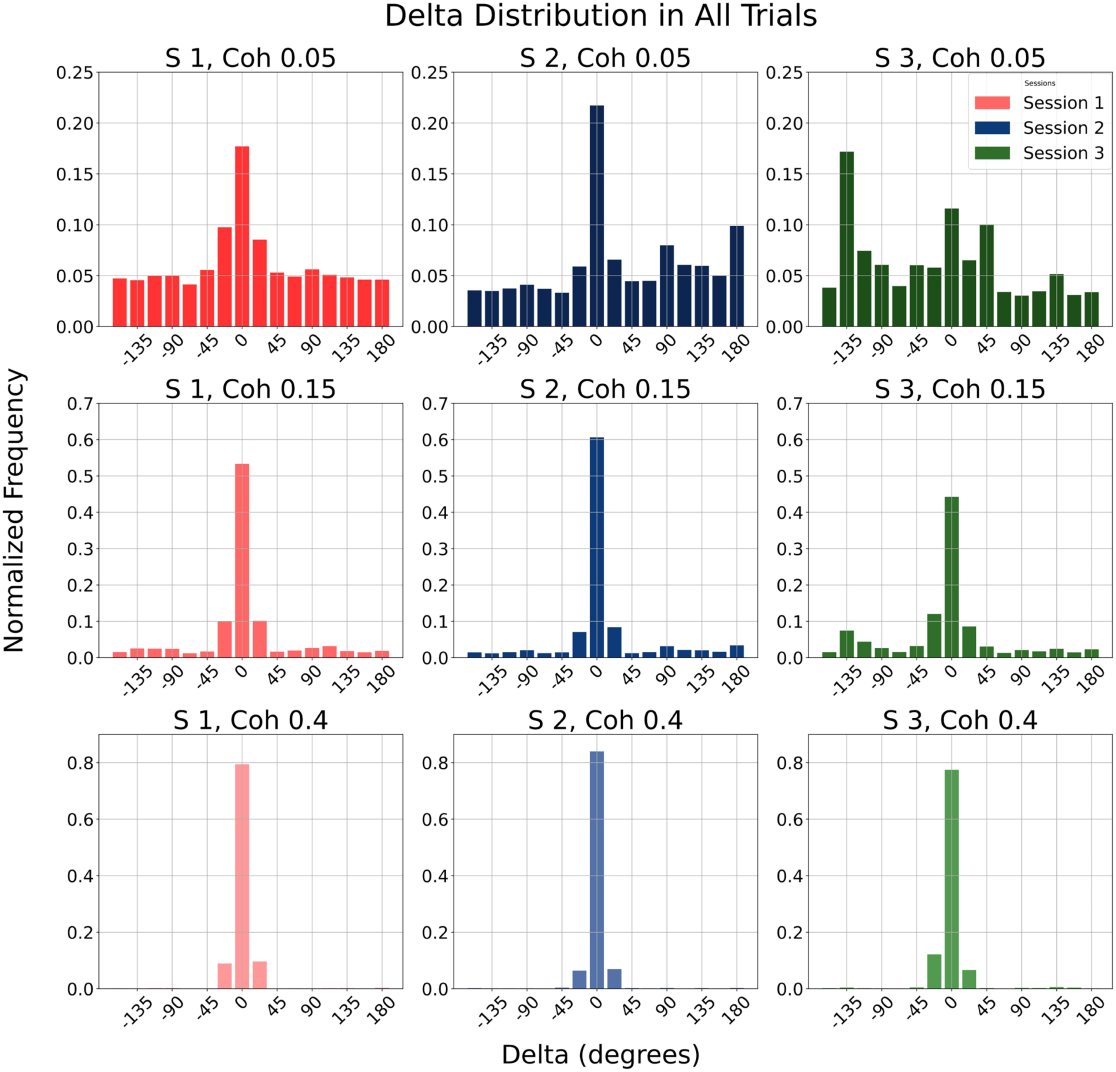
Delta distributions across sessions and coherence levels. Normalized histograms of angular error (delta) between reported and actual motion direction are shown for each session and motion coherence level. Rows correspond to coherence level (top, 5%; middle, 15%; bottom, 40%), columns correspond to session (left, unbiased Session 1; middle, rightward-biased Session 2 with bias at 0°; right, up-left–biased Session 3 with bias at −135°). Delta values are in degrees, computed as RDK motion direction minus reported direction, wrapped to the range (−180°, 180°). Positive values indicate counterclockwise errors, and negative values indicate clockwise errors; for example, in up-left–biased Session 3, a peak near −135° reflects responses around 0° (i.e., a clockwise shift away from the frequent −135° direction).

To examine systematic response patterns, we analyzed the distribution of direction errors (delta), defined as the angular distance between the true motion direction and the participant's response. Delta values close to zero indicate small errors (i.e., accurate estimates of motion direction), and larger values reflect increasing misperception. Delta distributions, pooled across participants, revealed that, as coherence increased, errors became more tightly centered on the correct direction (see Figure 3), consistent with improved motion visibility. At lower coherence levels (5% and 15%), delta values were more dispersed, indicating greater uncertainty. However, visual inspection of delta histograms revealed some non-trivial additional structure: in rightward-biased Session 2, where motion was predominantly to the horizontal right (0°), a prominent peak appeared near 180°, suggesting frequent misperception in the opposite direction with respect to RDK direction. Importantly, because this direction is also cardinal, we will later examine whether the apparent reversal reflects a cardinal bias rather than a genuine opposite-direction error.

In up-left–biased Session 3, where motion was biased toward −135° (upper-left oblique direction), delta values frequently clustered near −135°, which corresponds to a response direction around 0° (horizontal right). These patterns were especially pronounced in low-coherence conditions, pointing to systematic biases in motion estimation under uncertainty. This observation led us to examine the actual distribution of reported directions in error trials more directly.

#### Directional choice distributions

**Figure 4.**
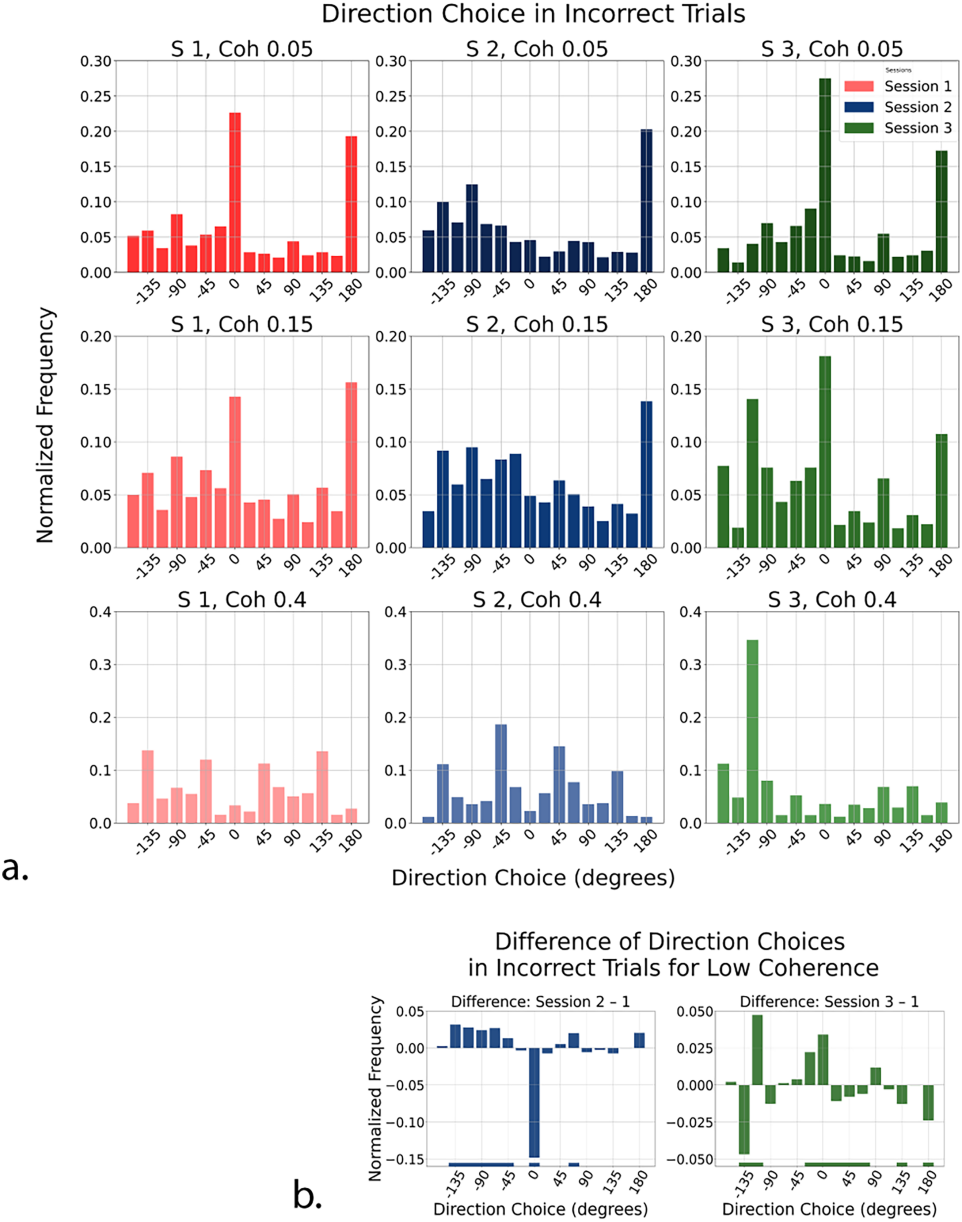
(**a**) Reported directions on incorrect trials across sessions and coherence levels. Shown are normalized histograms of direction choices on error trials, where the reported direction did not match the true motion direction. Rows indicate motion coherence (top, 5%; middle, 15%; bottom, 40%), and columns indicate session (left, unbiased; middle, Session 2 with rightward bias at 0°; right, Session 3 with upper-left bias at –135°). Directions are expressed in degrees, with 0° corresponding to horizontal rightward motion, negative values to the upper hemifield (e.g., −135° = upper-leftward), and positive values to the lower hemifield. (**b**) Difference in normalized frequency of reported directions between biased and unbiased sessions for incorrect trials: rightward-biased Session 2 minus unbiased Session 1 (*left*), and up-left–biased Session 3 minus unbiased Session 1 (*right*). Positive values indicate increased selection of that direction in the biased session compared with the unbiased baseline. The pronounced negative dip at 0° (rightward-biased Session 2) and −135° (up-left–biased Session 3) reflects a relative avoidance of the most frequent motion direction under uncertainty. Horizontal bars represent bins where the permutation test assessed a statistical difference with *p* < 0.01.

To gain further insight into the pattern of direction estimation errors, we examined participants’ direction choices on incorrect trials by plotting response histograms pooled across all participants (Figure 4). Across sessions and under low coherence conditions (5% and 15% only), these errors were not uniformly distributed but showed strong preferences for cardinal directions. Rightward (0°) was a frequent choice for error responses, followed by other cardinal directions, suggesting that, in the face of sensory ambiguity, perception tends to default to spatially familiar axes. These preferences are clearly visible in the unbiased Session 1.

However, these tendencies were modulated by session-specific direction statistics. In rightward-biased Session 2, where motion was predominantly horizontal right (0°), participants rarely chose 0° when they erred, instead frequently reporting 180°. At first glance, this shift resembled a repulsion effect, a response away from the true direction and in favor of the opposite one. However, this pattern was clarified through a comparison with the unbiased Session 1 (see below for details on the statistical analysis): Subtracting the unbiased Session 1 histogram from that of rightward-biased Session 2 revealed no significant increase in 180° responses. In other words, although 180° is a strong attractor in general, it was not more frequently chosen in that specific biased session. This suggests that the apparent repulsion was in fact a manifestation of a pre-existing bias toward cardinal directions, particularly horizontal, rather than a true effect of prior-driven repulsion.

In up-left–biased Session 3, where motion was biased toward −135°, participants similarly avoided the expected motion direction in their incorrect responses and frequently selected the rightward (0°) direction instead. This avoidance of the dominant direction was best captured, again, by subtracting from the normalized direction histograms of the biased sessions (Session 2 and 3) that of unbiased Session 1. These differences of histograms (see Figure 4b), computed by pooling incorrect trials from both 5% and 15% coherence conditions corresponding to low sensory reliability, revealed a clear dip around the frequently presented direction, especially where sensory input was most uncertain.

Importantly, this relative reduction at the most frequent direction was more pronounced in rightward-biased Session 2 than in up-left–biased Session 3. When the biased direction coincided with a cardinal axis (rightward, 0°), erroneous responses showed a clear redistribution away from that direction and toward alternative cardinal choices. In contrast, when the biased direction was oblique (−135°), the reduction in erroneous responses near the frequent direction was weaker and accompanied by increased selections of nearby bins, consistent with a stronger influence of intrinsic cardinal preferences. This asymmetry indicates that deviations from the frequent direction depend on the interaction between session-specific statistics and the cardinal structure of directional responses, rather than reflecting a uniform effect.

To statistically assess these shifts in response distributions, we implemented a permutation test on the frequency values (for direction estimates in error trials) within each angular bin. Specifically, we computed the observed frequency difference, defined as the mean difference in individual frequency values between biased and unbiased sessions, and compared it with the bootstrapped baseline distribution of frequency obtained by randomly assigning data to the unbiased and biased sessions and recomputing the frequency histograms and their difference, all this across 10,000 repetitions. The resulting *p* value was estimated numerically for each angular bin, by computing the probability that a bootstrap data sample (with random session labels) would have a larger frequency difference than the observed one. The horizontal colored bars at the bottom of Figure 4b highlight bins in which the *p* value for this statistical comparison was lower than 0.01.

Taken together, these findings suggest that, when motion direction is difficult to perceive, participants neither simply guess randomly nor select the most frequent option. Rather, their direction estimation systematically steers away from the most probable motion direction while continuing to cluster toward more cardinal, especially horizontal, axes. To better characterize these observations and test whether the avoidance dip actually reflects a systematic repulsion from the true motion direction and an attraction toward the opposite direction (as reported by Wu et al., 2021), we next analyzed the categorical relationship between participants’ responses and the actual motion direction.

#### Opposite-direction errors

To test whether these peaks reflected a repulsion effect from the true motion direction, we quantified the proportion of errors that fell 180° opposite the true direction. At low coherence (5%), opposite-direction errors were significantly more frequent in rightward-biased Session 2 (13.9% of errors) than in unbiased Session 1 (5.9%) and up-left–biased Session 3 (3.9%). This pattern held at 15% coherence (9.3% in rightward-biased Session 2 vs. ∼4% in the two others) and was statistically significant across all coherence levels (all *p* < 10⁻^10^). A GLMM predicting opposite-direction errors confirmed that these were more likely in rightward-biased Session 2 (β = 0.80, *p* < 0.001), suggesting indeed a repulsion effect away from the dominant motion direction and toward the diametrically opposite direction under uncertainty. However, such repulsion toward opposite was not observed for the other direction-biased session (up-left–biased Session 3), preventing a generalization of the previously reported effect (Wu et al., 2021).

#### Cardinal and horizontal biases

Across all sessions, participants displayed a persistent tendency to select cardinal directions, particularly 0° (horizontal right), −90° (upward), and 180° (leftward), when making incorrect responses (see Figure 4). To quantify this pattern, we computed the fraction of incorrect responses falling into cardinal bins (0°, ±90°, 180°), normalized by the number of bins in each category (four cardinal vs. 12 non-cardinal directions). We then subtracted the normalized fraction of non-cardinal errors to obtain a single bias score per trial. A GLMM predicting this cardinal-error tendency confirmed a significant bias in the unbiased Session 1 (intercept: β = 0.48, *p* = 0.009), indicating that, even in the absence of directional priors, participants disproportionately guessed along the cardinal axes. This tendency was significantly reduced in rightward-biased Session 2 (β = −0.51, *p* < 0.001), likely reflecting active suppression of rightward (0°) guesses under conditions of strong directional bias. A separate GLMM predicting 0°-directed errors showed that participants were significantly less likely to choose rightward motion in rightward-biased Session 2 (β = −1.40, *p* < 0.001), whereas, in up-left–biased Session 3, the tendency to revert to 0° increased marginally (β = 0.13, *p* = 0.065). These results suggest that the horizontal rightward direction holds a special status as a default guess under uncertainty: easily suppressed when a strong prior predicts rightward direction but otherwise dominant in the absence of clear sensory evidence.

Finally, we tested for vertical asymmetries in directional errors by fitting a GLMM predicting whether incorrect responses fell in the upper versus lower visual hemifield. Directions were classified as upper field if their angular value ranged from 0° to −180° (i.e., negative angles) and as lower field if from 0° to +180° (positive angles). For each trial, we computed the normalized fraction of errors falling into the upper hemifield (number of upper-bin errors divided by total bins per category, as in the cardinal error analysis), and subtracted the equivalent fraction for lower-bin errors to obtain a hemifield bias score. The model revealed a significant overall tendency toward upper-field responses (intercept: β = 0.38, *p* = 0.004), indicating that, across sessions, participants were more likely to misreport motion direction in the upper hemifield. This bias was further enhanced in the biased sessions (rightward-biased Session 2: β = 0.22, *p* < 0.001; up-left–biased Session 3: β = 0.28, *p* < 0.001), suggesting a spatial vertical asymmetry in directional estimation. Notably, this effect was independent of motion coherence, reinforcing the presence of non-uniform, directionally biased errors in motion estimation.

Together, these results show that, although accuracy improves with coherence, the structure of participants’ errors reflects systematic directional biases. Under uncertainty, participants did not consistently report directions close to the expected one (as predicted by the Bayesian attraction-to-prior hypothesis). Nor did they consistently exhibit repulsion away from the expected direction and toward the opposite one (Wu et al., 2021); yet, this pattern was observed in rightward-biased Session 2. Instead, in addition to a relative *avoidance* behavior related to the expected direction, direction estimation errors tended to default to cardinal, especially horizontal, directions. These cardinal biases remained robust even in the face of a strong manipulation of the contextual direction statistics. These findings challenge both prior-attraction and repulsion-toward-optimal models of perceptual inference for direction estimation in two dimensions, pointing instead to a more complex picture, where long-lasting directional preferences play a key role shaping motion perception under uncertainty, together with biases induced by the more recent experience.

#### Serial dependence analysis

In this study, our main objective was the analysis of the long-term adaptation of motion perception and eye movements to a statistical bias in visual motion direction, in line with recent publications (Carneiro Morita et al., 2025; Damasse et al., 2018; Darlington et al., 2018; Kowler et al., 2019; Wu et al., 2021). However, another branch of the literature (Cicchini et al., 2017; Fischer & Whitney, 2014; Zhang & Alais, 2020) in visual perception has reported interesting effects of short-term adaptation referred to as *serial dependence effects*. These prior findings suggest that perceptual decisions can be biased by recent experience, potentially leading to systematic errors or shifts in response patterns, based on the stimulus observed in the few previous trials. In the present work, serial dependence was examined to determine whether the response in a given trial was influenced by the RDK motion direction in the previous trial. If such an effect were present, it would indicate that participants integrated prior motion signals into their perception of the current stimulus.

In the unbiased Session 1, circular analyses provided no reliable evidence of serial dependence. A Watson–Williams one-way circular ANOVA, which is specifically appropriate for angular data that do not follow a normal distribution, revealed no significant modulation of perceptual error by the motion direction of the previous trial (*p* = 0.26), consistent across both Python and MATLAB implementations (Berens, 2009; Berens & Sinz, 2017). Visual inspection likewise showed participant-level means of delta values scattered around zero with error bars spanning tens of degrees. Together, these results indicate that, in the unbiased condition, perceptual reports were not systematically modulated by the motion direction of the previous trial, providing no evidence of either attractive or repulsive bias for trial-by-trial serial dependence. These results are illustrated in Supplementary Figure S1.

### Eye-movement results

**Figure 5.**
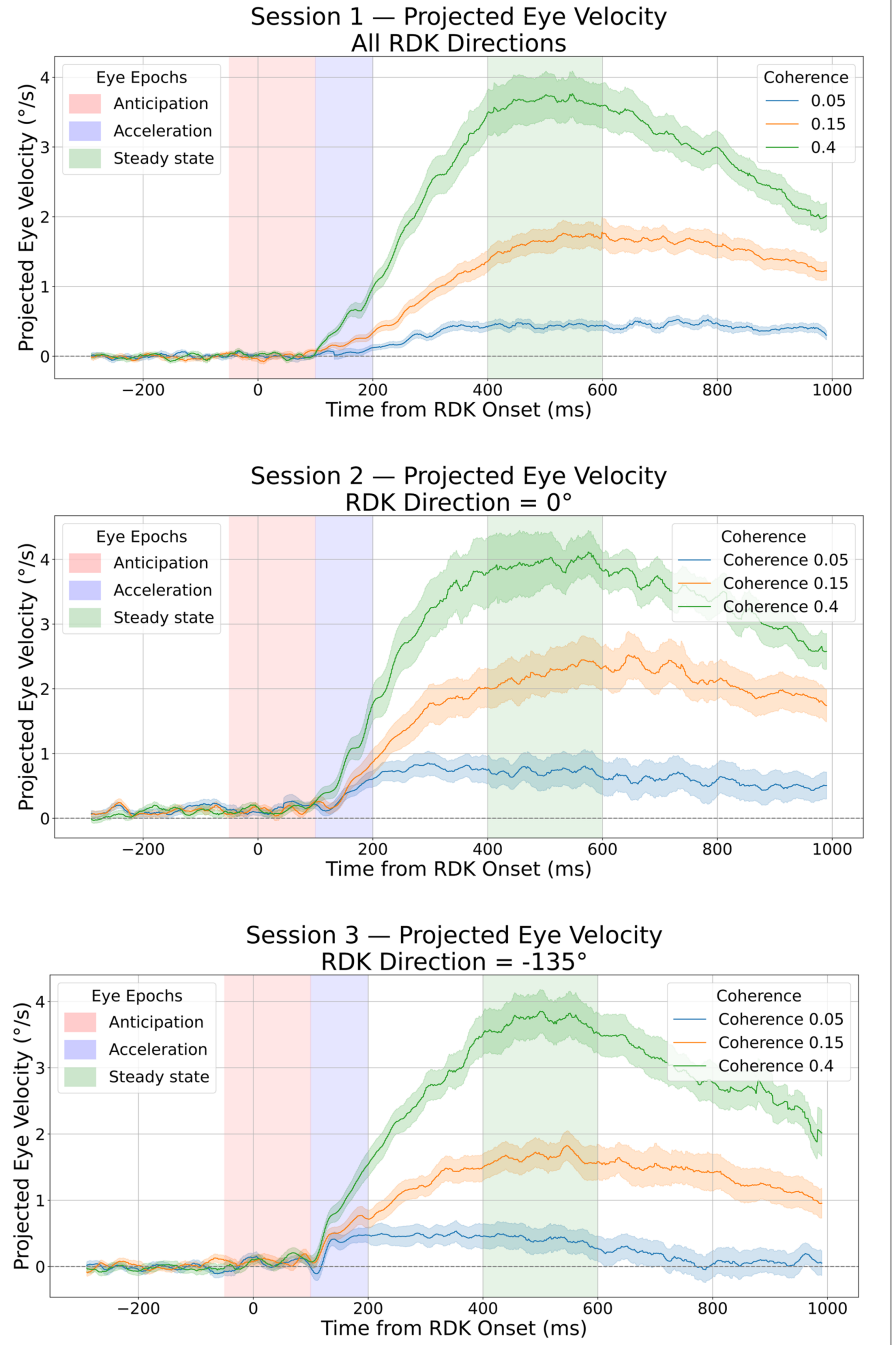
Projected smooth pursuit eye velocity across sessions and coherence levels. The time course of projected eye velocity is aligned to stimulus onset (0 ms) for each session. Line color indicates motion coherence (5%, 15%, 40%); shaded vertical bands denote analysis windows for anticipation (−50 to 100 ms), early acceleration (100–200 ms), and steady-state pursuit (400–600 ms). (*Top*) Unbiased Session 1 shows average pursuit responses across all motion directions. (*Middle*) Rightward-biased Session 2 shows pursuit responses for trials with RDK motion at 0°. (*Bottom*) Up-left–biased Session 3 shows pursuit responses for trials with RDK motion at −135°.

To visualize smooth pursuit behavior across conditions, we plotted the mean projected eye velocity for each session: unbiased Session 1, rightward-biased Session 2 (restricted to trials with 0° motion), and up-left–biased Session 3 (restricted to trials with −135° motion). Figure 5 provides an overview of the time course of mean eye velocity aligned with stimulus motion across different coherence levels and prior conditions.

#### Anticipatory eye movements reflect direction-specific adaptation to motion history

Our primary analysis of anticipatory velocity was focused on whether it increased when the same direction was repeatedly presented. To allow for direct comparison, we selected trials based on the direction of anticipation:

• 0° anticipation in rightward-biased Session 2 compared with 0° anticipation in unbiased Session 1
• −135° anticipation in up-left–biased Session 3 compared with −135° anticipation in unbiased Session 1

The distributions of individual anticipatory velocities projected onto 0° and −135° were tested for normality using the Shapiro–Wilk test (0°: *W* = 0.931, *p* = 0.145; −135°: *W* = 0.864, *p* = 0.008), indicating that parametric assumptions were met for the rightward comparison but not for the upper-left one. Statistical comparisons were performed at the subject level using paired *t*-tests, complemented by effect size estimates (Cohen's *d*), Bayesian *t*-tests (*BF*_10_), and Wilcoxon signed-rank tests when normality was violated. For rightward anticipation (rightward-biased Session 2 vs. unbiased Session 1), anticipatory velocity toward 0° increased significantly, *t*(20) = 3.88, *p* = 0.0009, *d* = 0.92, *BF*_10_ = 38.4. The Wilcoxon test confirmed this (*p* = 0.0019). In contrast, no significant change was observed for upper-left anticipation (up-left–biased Session 3 vs. Session 1), *t*(20) = 1.48, *p* = 0.154, *d* = 0.40, *BF*_10_ = 0.59, Wilcoxon *p* = 0.27.

**Figure 6.**
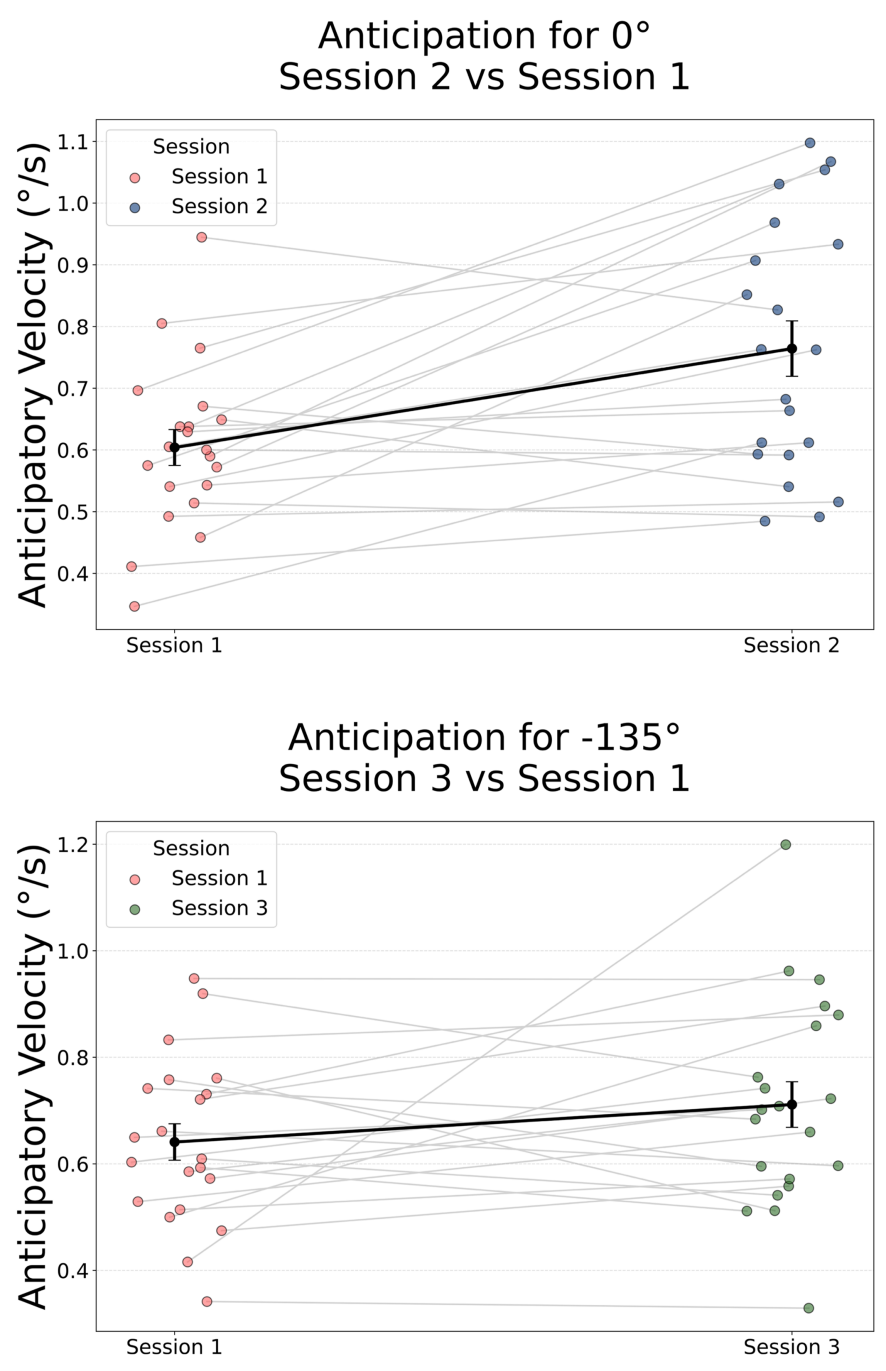
Anticipatory pursuit velocity for biased motion directions. Paired individual data and group means (±*SEM*) for anticipatory eye velocity in biased sessions compared to the unbiased baseline (Session 1). (*Top*) Anticipation eye velocity in the 0° direction in rightward-biased Session 2 in comparison with the unbiased session. (*Bottom*) Anticipation eye velocity in the −135°direction in up-left–biased Session 3 in comparison with the unbiased session. Each line connects the same observer across sessions.

Thus, although direction-specific anticipatory pursuit significantly increased in response to a frequently presented rightward stimulus in Session 2, no significant effect was observed toward the frequently presented upper-left motion in Session 3. This asymmetry suggests that anticipatory adaptation depends on the stimulus motion direction, with cardinal directions such as rightward motion (0°) eliciting more robust predictive eye movements than oblique directions (−135°) (Figure 6).

The absolute magnitude of anticipatory modulation was modest, as expected for anticipatory pursuit, but systematic. Importantly, because motion probability in our task was distributed across 16 possible directions, the observed modulation is comparable in scale to anticipatory effects reported in two-alternative designs (Wu et al., 2021) when accounting for the substantially higher uncertainty inherent to the present paradigm.

**Figure 7.**
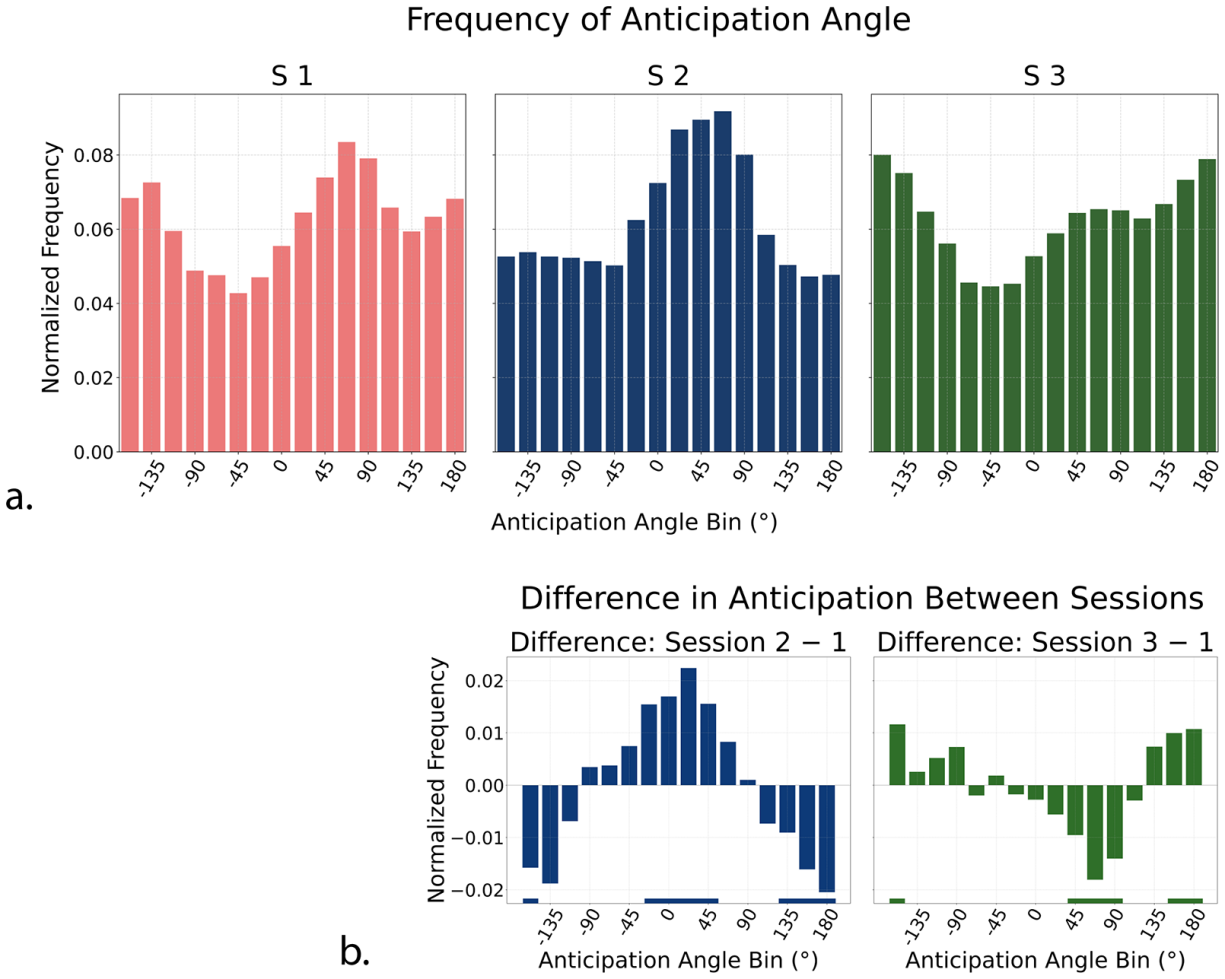
(**a**) Normalized frequency of anticipatory eye movement angles. Angles are binned into 22.5° steps. Zero indicates rightward motion; positive values correspond to downward/clockwise directions; and negative values indicate upward/counterclockwise directions. Rightward-biased Session 2 and up-left–biased Session 3 revealed distinct directional biases compared with the more uniform distribution in unbiased Session 1. (**b**) Difference in normalized anticipation angle frequency between biased and unbiased sessions. (*Left*) Rightward-biased Session 2 minus unbiased Session 1. (*Right*) Up-left–biased Session 3 minus unbiased Session 1. Angles are binned into 22.5° steps. Positive values indicate clockwise (downward) directions, and negative values indicate counterclockwise (upward), with 0° representing rightward motion. The difference in Session 2 – Session 1 shows a positive shift centered around 22.5°, consistent with rightward and slightly downward anticipation. The difference in Session 3 – Session 1 shows a negative dip for positive rightward direction and a positive shift to broad leftward direction. Horizontal bars represent bins where the permutation test assessed a statistical difference with *p* < 0.01.

We then addressed the question of whether the direction of anticipatory velocity could reflect a more general pattern of directional preferences, such as those related to the cardinal direction biases. To characterize these effects, we analyzed the distribution of anticipatory movement directions across 16 angular bins (each 22.5° wide). For each subject and session, we computed a normalized frequency histogram based on the angle of the anticipatory eye velocity vector. Then, we pooled the individual frequency histograms in a single group histogram (Figure 7a). In the baseline unbiased Session 1, anticipatory directions were weakly biased toward rightward and downward angles. In rightward-biased Session 2, this bias to the right became more pronounced. In up-left–biased Session 3, however, anticipatory directions were more diffusely distributed across leftward bins, lacking a clear peak near the repeated −135° direction. To isolate the effect of the experimental manipulation of direction probability from general long-term biases, we subtracted each subject's unbiased Session 1 histogram from their histograms in biased Sessions 2 and 3 and then pooled again the resulting histograms across participants, to illustrate the result in Figure 7b. These frequency-difference plots revealed a focused increase in anticipatory movements toward 0° in rightward-biased Session 2, but only a broad and inaccurate peak of anticipation (centered around 180° rather than −135°) in up-left–biased Session 3. Similar to direction choice differences, permutation tests were computed for anticipation differences and are illustrated in Figure 7b (horizontal bars on the bottom).

Together, these results show that anticipatory pursuit aligns to the statistics of prior motion, but this adaptation is direction dependent, favoring again cardinal (horizontal) directions. These findings set the stage for examining whether such early predictive behavior is also reflected in subsequent stages of pursuit—specifically, initial pursuit acceleration and steady-state tracking.

Finally, in addition to the apparent global disagreement between the pattern of anticipatory eye velocity and direction estimation biases in biased sessions, we also computed, in all sessions and for all coherence levels, the trial-by-trial correlation between anticipation direction and direction estimates. The Pearson's correlation coefficient was non-significant (*r* = 0.00 to 0.06 across conditions; all *p* > 0.10).

#### Initial pursuit acceleration reflects general sensorimotor gain adaptation

To examine how prior experience with motion direction influences the early sensorimotor response, we analyzed pursuit mean acceleration during the early visually guided phase following pursuit onset (within the open loop). This phase reflects the buildup of visually driven tracking and is thought to be modulated by both sensory reliability and prior exposure to specific motion properties (Darlington et al., 2018; Deravet et al., 2018). According to the Bayesian integration hypothesis for eye movement control, we expected a more robust tracking response in the frequently presented direction (prior facilitation) in biased sessions than in the same direction for the unbiased Session 1. We also expected that the prior facilitation would be relatively more important for low coherence stimuli—that is, when the sensory evidence was weaker.

To capture how acceleration varied with sensory reliability and exposure to a direction bias, we averaged projected acceleration values across trials per subject, session, and coherence level. These subject-level means were submitted to repeated-measures ANOVAs with Session and Coherence as within-subject factors (Subject as the repeated factor). We first compared all trials in every 16 possible motions in the unbiased Session 1 with direction-specific (biased) trials in rightward-biased Session 2 (rightward, 0°) and up-left–biased Session 3 (upper-left, −135°). The ANOVA revealed significant main effects of Session: Session 1 versus Session 2, *F*(1, 20) = 13.76, *p* = 0.0014; Session 1 versus Session 3, *F*(1, 20) = 34.43, *p* < 0.00001. This finding indicates overall higher acceleration in the biased sessions compared with the unbiased baseline. There were also strong main effects of Coherence (*F* > 61, *p* < 10⁻^8^), showing that acceleration increased with motion coherence. No significant Session × Coherence interactions were observed (*p* > 0.16).

**Figure 8.**
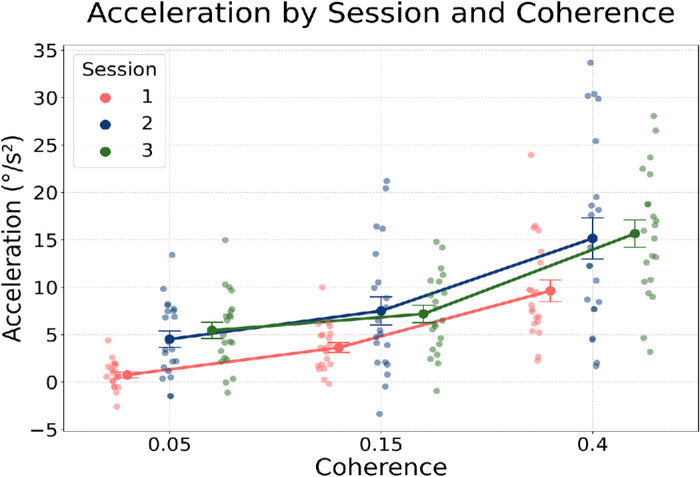
Early acceleration by session and coherence level. Acceleration was computed as the difference, per unit time, in projected eye velocity between 200 ms and 100 ms after stimulus onset. All trials were included in unbiased Session 1, whereas for Sessions 2 and 3 only trials with motion directions at 0° (rightward) and −135° (upper-leftward), respectively, were selected. Error bars reflect between-subject *SEM*.

To provide a direction-matched comparison, we additionally ran the same repeated-measures ANOVA using only trials corresponding to the biased directions—that is, trials with 0° motion in Sessions 1 and 2, and with −135° motion in Sessions 1 and 3. This selection ensured a cleaner match across conditions, although the number of trials in unbiased Session 1 for these specific directions was limited. In this more restricted analysis, acceleration remained significantly higher for the rightward bias (Session 1 vs. Session 2), *F*(1, 20) = 5.65, *p* = 0.028, but not for the upper-left bias (Session 1 vs. Session 3), *F*(1, 20) = 1.36, *p* = 0.26. Coherence remained a strong main effect in both comparisons (*F* > 32, *p* < 0.001), with no significant interactions between Session and Coherence. These results indicate that early pursuit acceleration increased reliably when motion statistics were biased, especially for the rightward (cardinal horizontal) direction. (Figure 8).

As expected, sensory reliability also modulated pursuit acceleration: In all sessions, acceleration increased significantly with RDK coherence (Figure 8). However, the absence of a significant interaction between Session and Coherence prevents drawing firm conclusions about whether direction priors and sensory likelihoods combine in a reliability-weighted manner at this early processing stage. These findings suggest that the oculomotor system increases sensorimotor responsiveness globally in response to biased motion statistics. This pattern is consistent with theoretical accounts of gain control in smooth pursuit (Darlington et al., 2018) and with Bayesian models that allow for tuning-specific learning or partial adaptation, to statistical structure.

#### Steady-state pursuit tracks sensory evidence, not motion history

Finally, we analyzed steady-state pursuit, the later phase of smooth tracking in which sensory input is expected to dominate over prior expectations. If the system transitions from prior-driven to likelihood-driven control over time (Rubinstein et al., 2024), this component should mainly reflect the fidelity of visual motion processing rather than learned bias.

We computed the mean projected eye velocity between 400 ms and 600 ms after stimulus onset—a time window included in the closed-loop period and chosen to capture stable pursuit behavior. We first compared all trials in unbiased Session 1 with direction-specific (biased) trials in rightward-biased Session 2 (rightward, 0°) and up-left–biased Session 3 (upper-left, −135°). The repeated-measures ANOVA revealed a strong main effect of Coherence for Session 1 versus Session 2, *F*(2, 40) = 151.36, *p* < 0.001, and for Session 1 versus Session 3, *F*(2, 40) = 139.75, *p* < 0.001, indicating that pursuit velocity increased systematically with motion coherence. The main effect of Session was not significant for Session 1 versus Session 2, *F*(1, 20) = 3.23, *p* = 0.088, or for Session 1 versus Session 3, *F*(1, 20) = 0.22, *p* = 0.65, and there were no Session × Coherence interactions (*p* > 0.10).

**Figure 9.**
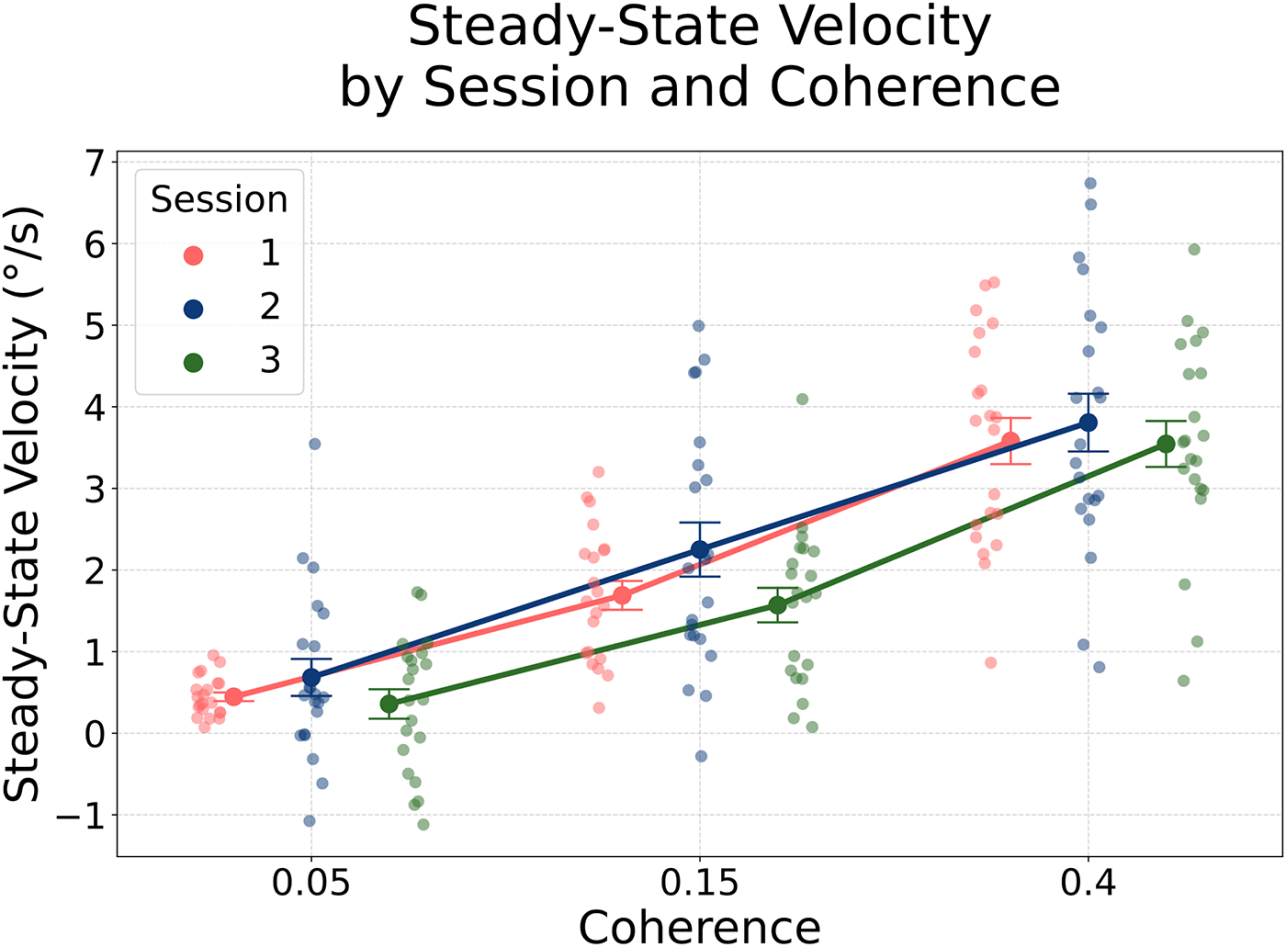
Steady-state pursuit velocity across sessions and coherence levels. Steady-state velocity was computed as the mean projected eye velocity between 400 ms and 600 ms after RDK onset, aligned with the true motion direction. All trials were included in unbiased Session 1, whereas for Sessions 2 and 3 only trials with motion directions at 0° (rightward) and −135° (upper-leftward), respectively, were selected. Each point reflects a subject-level mean for a given coherence level and session. Error bars represent between-subject *SEM*.

To ensure a direction-matched comparison, we repeated the analysis including only trials corresponding to the biased directions: toward 0° motion in Sessions 1 and 2 and toward −135° motion in Sessions 1 and 3. This restriction provided a cleaner correspondence between bias and baseline directions, although the number of trials in Session 1 was still limited. The pattern remained consistent: Coherence continued to exert a strong influence on steady-state velocity (*t* > 2.7, *p* < 0.02 for higher vs. lower coherence), whereas no reliable session differences emerged (*p* > 0.08). Together, these results indicate that steady-state pursuit primarily reflected the quality of sensory motion input, with higher coherence producing faster and more stable eye velocity, and little or no modulation by prior exposure to specific motion directions (Figure 9).

In addition, we calculated the trial-by-trial Pearson correlation between the steady-state eye velocity direction and the perceptual direction estimate to assess whether perceptual and oculomotor responses were aligned within individual trials. Across all sessions, the correlation between steady-state angle and perceived direction was small but consistently positive, strengthening with increasing motion coherence. In unbiased Session 1, correlations rose from *r* = 0.04 (*p* = 0.019) at 5% coherence to *r* = 0.43 (*p* < 0.001) at 40%. A similar pattern was observed in rightward-biased Session 2 (*r* = 0.05, 0.06, and 0.13 for 5%, 15%, and 40% coherence, respectively; all *p* ≤ 0.005) and up-left–biased Session 3 (*r* = 0.12, 0.11, and 0.34, respectively; all *p* < 0.001). These results indicate that the steady-state pursuit direction closely tracked perceptual estimates, particularly when sensory evidence was strong.

In conclusion, steady-state pursuit was strongly modulated by motion coherence, with higher coherence levels yielding faster and more reliable tracking—consistent with improved visual motion signals. However, there were no differences between sessions, indicating that prior exposure to specific directions had no consistent influence by this stage.

These results replicate and extend findings from Rubinstein et al. (2024), who showed that prior influence on pursuit decays over time, giving way to sensory-driven tracking. Our data support the view that the oculomotor system transitions from prior- to likelihood-dominated control over time. By approximately 400 ms post-stimulus onset, pursuit appears to rely predominantly on sensory evidence (likelihood), not on the experience-based direction prior, consistent with Bayesian models of dynamic sensorimotor integration (Bogadhi et al., 2013; Körding & Wolpert, 2004; Orban De Xivry et al., 2013).

#### Serial dependence effect in unbiased session

To determine whether anticipatory eye movements were influenced by the motion direction of the previous trial (Goettker & Stewart, 2024; Zhang & Alais, 2020), we analyzed data from the unbiased Session 1 using a linear mixed-effects model. The model predicted anticipation angle on trial *n* based on the global motion direction on trial *n* – 1, with random intercepts by subject.

The analysis revealed no significant relationship between the motion direction of the previous trial and the anticipation angle on the current trial (β = 0.017, *SE* = 0.010, *z* = 1.65, *p* = 0.099) (see Supplementary Figure S2). This indicates no reliable evidence that participants’ eye movements were biased in the direction of the previous stimulus, suggesting an absence of short-term serial dependence effects on anticipatory oculomotor behavior under these task conditions.

## Discussion

### Summary of key findings

In this study, we investigated how uncertain motion cues are combined with expectations about motion direction to control motion perception and smooth eye tracking across different directions in the two-dimensional space. In the following sections, we discuss the main findings and the perspectives open for future research.

### Direction estimation biases in a direction-biased environment

Our results reveal a striking inhomogeneity in how perceptual decisions about motion direction are shaped by prior experience and long-term structural biases. In sessions where a specific motion direction was overrepresented (0° in Session 2 and −135° in Session 3), participants’ motion estimates were not *attracted* toward the frequent direction, as would be predicted by the classical Bayesian attraction-to-prior hypothesis under uncertainty. Instead, they tended to avoid the expected direction with low coherence stimuli—that is, when the sensory likelihood was weak. In addition, we could systematically observe a relative preference toward cardinal directions. Incorrect reports often aligned with the horizontal or (to a lesser extent) vertical axes, regardless of the session-specific prior.

This avoidance pattern does not align with the prediction of a prior-attraction bias of simple Bayesian integration models (but see further discussion below), nor does it reflect repulsion toward the opposite direction. Instead, it suggests a hybrid regime in which deeply rooted directional biases, such as the well-established preference for cardinal directions in orientation and direction perception (Andrews & Schluppeck, 2000; Appelle, 1972; Girshick et al., 2011; Tomassini, Morgan, & Solomon, 2010), interact with shorter-term statistical biases to shape responses. The consistent fallback to 0° in both unbiased Session 1 and up-left–biased Session 3 and the shift toward 180° in rightward-biased Session 2 underscore the strength and persistence of these internal priors (with some session-specific readjustments).

Our results echo previous findings showing that, under high sensory uncertainty, perception often defaults to familiar, high-probability directions in the naturalistic environment, rather than relying exclusively on recent stimulus history (Geisler, 2008; Knill & Pouget, 2004; Simoncelli & Olshausen, 2001; Straub & Rothkopf, 2021). This may indicate that perceptual priors operate at multiple time scales, with long-term environmental regularities dominating over short-term exposure, particularly when sensory input is ambiguous or weak.

### Avoidance versus repulsion of direction estimates

Our findings diverge from both classical attraction-to-prior predictions and previous demonstrations of perceptual repulsion. In our unbiased Session 1, where motion directions were uniformly distributed, participants did not show evidence of repulsion away from the current or the recently observed directions (e.g., serial effect analysis). Perceptual errors did not systematically deviate from prior stimuli, nor did they cluster opposite to them. In Sessions 2 and 3, where motion was biased toward a specific direction (0° or −135°), participants did not exhibit repulsion, either; that is, they did not consistently report motion in the opposite direction to the prior. In rightward-biased Session 2, the pattern of perceptual errors could easily be misinterpreted in that sense: Participants often responded around 180°, the direction opposite to the frequent 0° motion. However, this shift likely reflects a defaulting to horizontal (cardinal) axes under uncertainty, not a targeted repulsion from 0°, as suggested by the fact that the normalized histograms of inaccurate direction choices are not significantly different for the 180°-centered bin (e.g., Figure 4). Similarly, in up-left–biased Session 3, participants avoided the −135° direction but showed no consistent bias toward +45°, the geometric opposite. Instead, they again clustered toward 0°, a familiar and stable reference direction.

Together, these patterns do not confirm the established observation of repulsion as found in Bae and Luck (2022) or Chetverikov and Jehee (2023), where participants’ percepts were systematically displaced 180° away from frequently presented or previously seen stimuli. In those studies, repulsion emerged even without explicit experimental biasing and was often interpreted as a distortion in sensory representation or decoding. In contrast, our participants avoided the most frequent direction in the biased sessions but did not systematically shift toward any specific alternative, particularly not toward the direction opposite to the frequent one. This pattern differs from the findings (or rather the interpretation of those findings) of our previous study (Wu et al., 2021), where repulsion toward the opposite direction of the frequent one was reported. The limiting factor in that study is that only horizontal motion directions (right/left) were tested, leading to a deep confound between avoidance of the expected direction and repulsion toward the opposite direction.

What, then, explains this hybrid pattern? Under low sensory reliability, participants showed neither attraction to the experimentally induced prior (as predicted by Bayesian integration) nor repulsion from it. Instead, they seemed to avoid the frequent direction and revert to internal structural priors, in this case, cardinal directions. This suggests a distinct mode of processing, where long-term environmental statistics override recent experience in shaping the internal model of the environment and hence perception. In any case, further work, at the theoretical and experimental level is necessary to clarify the underlying mechanisms. In particular, the specificity of the moving stimulus properties should be investigated to assess the generalizability of these results.

Repulsion-like behaviors have previously been reported, and they may not completely contradict Bayesian inference. Jazayeri and Movshon (2007) showed that perceptual repulsion can emerge from the decoding stage of Bayesian perceptual inference, depending on the task specificities. When neural population responses are broad and overlapping, especially near category boundaries or under ambiguity, optimal decoding can shift percepts away from the boundary direction to maximize discriminability. Although their study focused on categorical estimation, similar mechanisms may apply in our continuous direction estimation task, especially under uncertainty.

An interesting research line (see Fritsche, Spaak, & de Lange, 2020; Hahn & Wei, 2024; Wei & Stocker, 2015) offers a related explanation grounded in a general model encompassing neural efficient coding (Barlow, 1961) and Bayesian decoding. These studies proposed that sensory encoding is shaped by natural stimulus statistics. As a consequence, the sensory likelihood function represented by early neuronal populations could be asymmetric, and its combination with a prior could yield posteriors biased away from, rather than toward, the prior. Some authors refer to this phenomenon as “anti-Bayesian,” although it remains fully consistent with normative Bayesian principles. However, note that the previously reported examples of perceptual biases away from the prior refer usually to small deviations (∼few degrees) from the expected response (typically one of the cardinal orientations), and they were mostly documented in experiments addressing the participants’ direction discrimination performance (e.g., Rauber & Treue, 1998). In contrast, our participants were asked to estimate RDK motion direction, and the repulsion effects induced by the experimental direction biases in Sessions 2 and 3 had a much larger size (e.g., above 90°). At this stage, it is difficult to integrate our results in the above-mentioned theoretical framework, but the suggestion that neuronal encoding properties in early visual areas may display efficient coding properties, in line with natural motion statistics, seems coherent with our robust biases for cardinal directions. Future studies are needed to further elucidate the common theoretical ground proposed by Hahn and Wei (2024) and extend it to all of the different perceptual biases observed across time scales.

Overall, this theoretical framework suggests that attraction and repulsion are not contradictory but rather reflect different outcomes of a shared inferential process. Depending on the structure of internal representations and the reliability of sensory input, perception may either lean into the prior or shift away from it.

### Smooth pursuit eye movements as dynamic indicator of Bayesian integration

In contrast to perception, smooth pursuit eye movements showed signs of attraction behavior to repeated motion directions, although with important asymmetries. It is necessary to underline that already in the unbiased Session 1, and similar to inaccurate direction estimates, the angular distribution of anticipatory eye velocity was strongly non-homogeneous across directions. As a consequence, the main effects of the experimental direction bias became apparent only when we considered the difference between anticipation in the biased and the unbiased sessions. We should thus interpret the anticipation results as *relative* to the pattern observed in unbiased Session 1, which serves as baseline. In Session 2, where motion was biased toward 0°, anticipatory pursuit velocity significantly increased with anticipation peaking around the expected direction but also drifting slightly downward and rightward, as confirmed by direction histograms (Figure 7). In Session 3, biased toward −135°, no significant increase in anticipatory velocity was observed, but histogram analyses revealed a general broad shift of anticipation toward the left hemifield. These patterns suggest that predictive oculomotor anticipation is influenced by recent motion statistics but is also more precise and reliable when aligned with cardinal directions. Overall, this result is consistent with previous findings that anticipatory pursuit reflects cognitive expectations and is shaped by both environmental regularities and exposure history (Kowler et al., 2019; Kowler & Steinman, 1979a; Kowler & Steinman, 1979b; Santos & Kowler, 2017; Wu et al., 2021). Compared with two-alternative forced-choice paradigms (e.g., Wu et al., 2021), anticipatory pursuit modulation in the direction-biased sessions of the present task was reduced in magnitude, consistent with the distribution of direction probability across multiple motion directions and thus the higher directional uncertainty.

Early visually guided pursuit, measured as acceleration in the 100- to 200-ms window after stimulus onset, showed reliable increases for both biased directions. This suggests that, during the open-loop phase, the oculomotor system integrates sensory information with the recent motion statistics, in line with models proposing Bayesian combination of priors and likelihoods weighted by reliability (Darlington et al., 2018; Deravet et al., 2018; Park, Kim, Kim, & Lee, 2023). Yet, the effect was stronger in Session 2, where the bias was toward the cardinal (rightward) direction and weaker in up-left–biased Session 3, indicating that cardinal motion remains dominant even at this early stage. However, the signature of Bayesian integration was not observable in the late phase of pursuit. Steady-state pursuit, measured 400 to 600 ms post-RDK onset, was influenced solely by sensory coherence, with no modulation by direction bias. This observation aligns with findings from Rubinstein et al. (2024), who showed that prior influence decays over time as pursuit becomes increasingly dominated by the ongoing visual signal. Their study also revealed that, although the pursuit system flexibly adapts to motion direction statistics, it does so in a sub-optimal way in terms of adherence to Bayesian integration predictions.

Altogether, the pursuit results support a layered framework of predictive oculomotor processing. Anticipatory pursuit reflects prior expectations shaped by recent statistics and long-term directional biases, expressed as a preparatory motor bias that emerges before sensory evidence from the current trial is available. Early acceleration reflects a closely related but distinct process, in which these expectations interact with incoming motion signals to modulate visuomotor gain, consistent with proposals that expectation scales the transformation from sensory evidence to motor output (e.g., Darlington et al., 2018). During the later closed-loop phase, pursuit behavior is driven primarily by immediate sensory evidence (as well as extra-retinal self-motion-related cues), with little influence of prior direction bias. This dynamic progression matches Bayesian models of motion tracking in which the relative contribution of priors and likelihood evolves over time, allowing predictive influences to dominate early stages of pursuit while sensory evidence progressively constrains behavior later on (Bogadhi et al., 2011; Bogadhi et al., 2013; Kowler et al., 2019; Montagnini et al., 2007; Orban De Xivry et al., 2013; Rubinstein et al., 2024).

### Relations between perception and pursuit

Our results show that, although both perception and pursuit are influenced by prior experience and uncertainty, they rely on distinct computational strategies and exhibit different sensitivities to environmental and experimental manipulations. Pursuit, particularly in its anticipatory and early acceleration phases, adapted flexibly to repeated motion directions in an attraction-to-prior mode, especially when the bias aligned with cardinal axes.

In contrast, perception remained anchored to long-standing directional preferences, showing evidence of avoidance of, rather than attraction to, the frequent direction. The present dissociation between eye movement behavior and perceptual responses suggests that perception and pursuit do not simply differ in sensitivity but instead may operate based on different priors, or apply distinct decoding or decision processes to similar priors. This dissociation echoes previous reports, reviewed, for example, in Spering and Montagnini (2011), and in the works of Spering and Carrasco (Carrasco & Spering, 2024; Spering & Carrasco, 2015), as well as, more specifically, previous findings of our group (e.g., Wu et al., 2021). It also supports the idea that the oculomotor and the perceptual systems process expectations along partly separate pathways. Although pursuit reflects continuous motion integration and is sensitive to recent trial statistics, perceptual decisions appear to rely more heavily on longer-term environmental knowledge and structural constraints in neural representation. These systems may thus implement predictive strategies at different levels of abstraction and on different time scales.

Importantly, both systems showed signs of direction-specific modulation: Anticipatory pursuit increased selectively for 0° but not for −135°, and perceptual avoidance was stronger for 0° in rightward-biased Session 2 than for −135° in up-left–biased Session 3. This suggests that, even when both systems access the same stimulus statistics, their internal weighting and decoding are direction dependent, possibly reflecting natural anisotropies in motion encoding and processing.

Taken together, our findings argue against a unified Bayesian decoder that governs both action and perception. Instead, they point to a layered architecture in which shared priors are accessed and integrated differently depending on the function of the system, such as predictive tracking versus categorical estimation, and on its access to sensory and motor feedback.

Finally, on a more methodological note, encouraging participants to track global RDK motion with their gaze (rather than maintain fixation) could have affected motion direction perception. For example, the information about one own's eye displacement, or eye velocity direction, could be used as a cue to estimate RDK motion direction. Although we have not directly addressed this possibility, we consider that it could not undermine the validity of the present results, which are mainly related to (a) the effect of prior information upon visual motion perception under high-uncertainty—that is, when tracking eye movements are very weak and variable; and (b) the relation between perceptual biases and biases of anticipatory eye velocity, two behaviors that were not significantly correlated on the trial-by-trial basis.

## Conclusions and future directions

This study contributes to the growing evidence that predictive processing is system specific and shaped by multiple interacting priors. By examining smooth pursuit and perceptual estimation under the same stimulus conditions (variable coherence RDK stimuli moving across all directions in the two-dimensional plane), we showed that, although pursuit flexibly aligns with recent statistics, perception remains dominated by entrenched directional biases and actively avoids the most frequent directions under ambiguity. These results highlight the layered and context-sensitive nature of inference in the brain.

Future research should continue to explore how perceptual and motor systems represent and update priors and whether these priors are shared, transformed, or independently constructed. Computational modeling, combining population coding and probabilistic inference, will be crucial to explaining the observed dissociations. Testing these processes under varying task demands, in naturalistic contexts, or across development and pathology may further clarify how predictive mechanisms are shaped and constrained.

In line with the legacy of Eileen Kowler's work, our findings reaffirm the value of integrating eye movement research into broader questions about inference, expectation, and the structure of visual cognition. Pursuit continues to provide a unique, dynamic expression of internal beliefs, and, when studied alongside perception, reveals the complexity of predictive behavior in uncertain environments.

## Supplementary Material

Supplement 1
