# Supplementary figures and images for: Motion direction biases around the clock: Learned and in-built direction priors pull perception and pursuit apart
Source: J Vis. 2026 Mar 23;26(3):11. doi: 10.1167/jov.26.3.11 (PMC13020133; doi:10.1167/jov.26.3.11)

## Supplementary materials

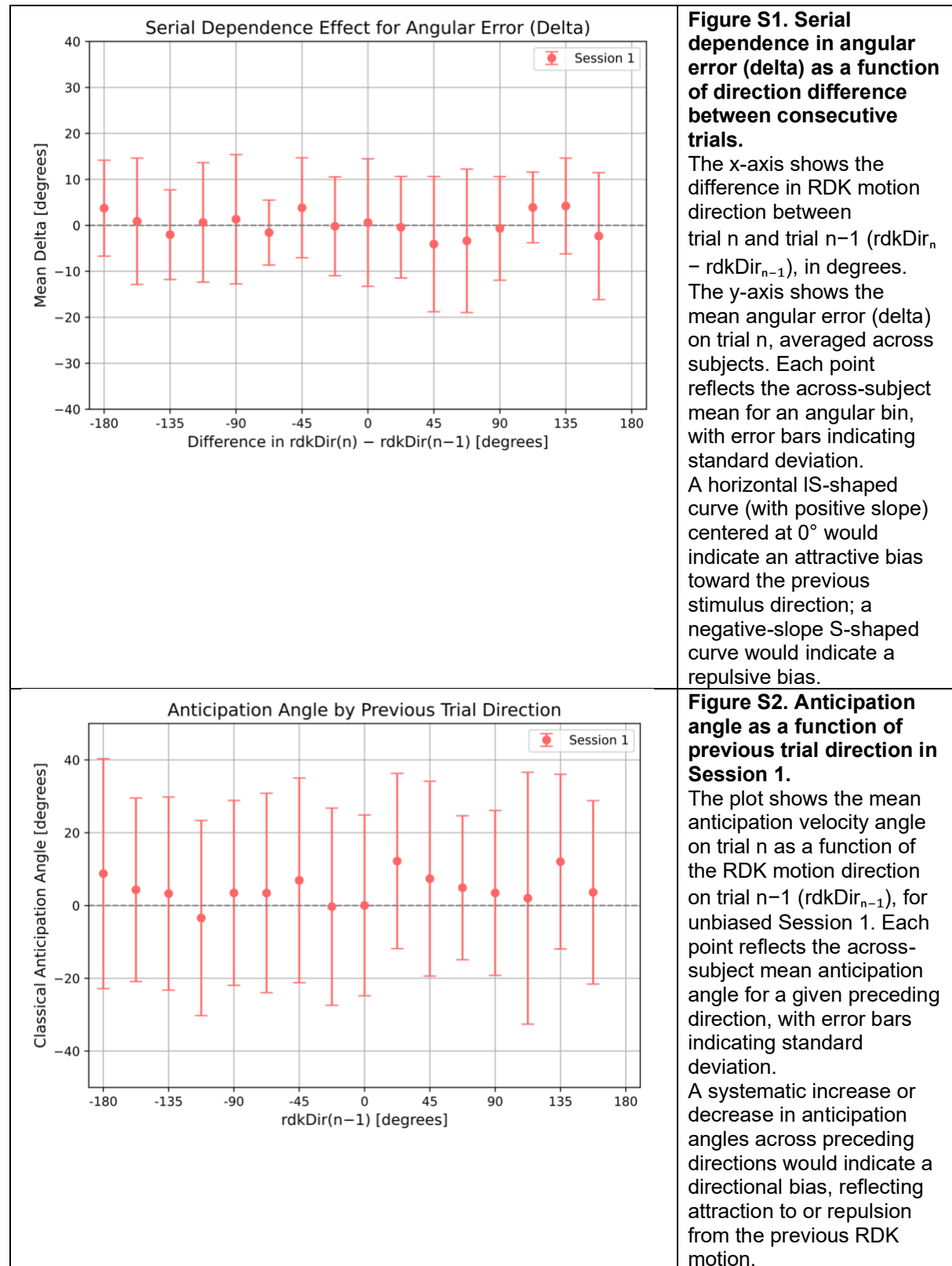

Supplement: Supplement 1 [file jovi-26-3-11_s001.pdf]
